# Interventions to Support the Mental Health of Frontline Healthcare Workers During the COVID-19 Pandemic: A Systematic Review

**DOI:** 10.3390/ijerph23081082

**Published:** 2026-08-19

**Authors:** Rosechelle M. Ruggiero, Hetal J. Patel, Carol S. North, Traci N. Adams

**Affiliations:** 1 Division of Pulmonary and Critical Care Medicine, University of Texas Southwestern Medical Center, 5323 Harry Hines Blvd, Dallas, TX 75390, USA; rosechelle.ruggiero@utsouthwestern.edu (R.M.R.);; 2 Department of Psychiatry, University of Texas Southwestern Medical Center, Dallas, TX 75390, USA

**Keywords:** COVID-19, mental health, frontline healthcare workers, interventions, psychological interventions, mental health interventions, mental health support programs

## Abstract

**Highlights:**

**Public health relevance—How does this work relate to a public health issue?**

- COVID-19 was a public health issue affecting the physical and mental health of billions of people.
- Interventions to support front-line healthcare worker mental health can be implemented by public health systems.

**Public health significance—Why is this work of significance to public health?**

- Preparation for the next pandemic should begin now.
- A review of effective interventions to support the mental health of front-line workers can inform pandemic preparation efforts.

**Public health implications—What are the key implications or messages for practitioners, policy makers and/or researchers in public health?**

- This systematic review suggests that an established disaster mental health (MH) response framework may be useful in directing the design of MH responses to front-line (FL) healthcare workers (HCWs) in future pandemics.
- Planning for the MH of FL HCWs in the next pandemic can begin now within the framework of the disaster mental health literature, offering screening for psychopathology and connection with formal psychiatric services for full evaluation and provision of MH care, as well as supportive care with mindfulness interventions, crisis-oriented counseling, psychoeducation, and psychological first aid.

**Abstract:**

The COVID-19 pandemic led to distress and an increased prevalence of psychopathology among front-line (FL) healthcare workers (HCWs). A synthesis of the available data on the effectiveness of MH interventions among FL HCWs is essential to inform an MH plan for FL HCWs in future pandemics. The objective of this systematic review is to examine interventions used to support the MH of FL HCWs during the COVID-19 pandemic. All studies published between 1 January 2020 and 1 March 2026 that reported on MH interventions among FL HCWs during the COVID-19 pandemic were identified using PubMed, PsycINFO, Scopus, EMBASE, Cochrane Database of Systematic Reviews, Google Scholar, Medline, and Web of Science. Studies published in peer-reviewed journals that reported data on interventions to support the MH of FL HCWs were included. The study was conducted using the approach by the PRISMA guidelines. The search generated 1450 records, of which 309 were reviewed at length, and 153 studies (N = 153) were included. Interventions in these studies included identification of risk for psychopathology and linkage to mental health care (n = 3), PE/PFA (n = 44), psychotherapy (n = 26), mindfulness programs (n = 58), medications (n = 5), and multiple/combined psychosocial support interventions (n = 17). The majority of studies found their interventions to be beneficial, largely based on user satisfaction scores and symptom screening scores. Importantly, symptoms in the vast majority of control arms also improved over time, though not to the degree that symptoms in intervention arms improved. This systematic review suggests that an established disaster MH response framework may be useful in directing the design of MH responses to FL HCWs in future pandemics, offering screening for psychopathology and connection with formal psychiatric services for full evaluation and provision of MH care, as well as supportive care with mindfulness interventions, crisis-oriented counseling, PE, and PFA.

## 1. Introduction

The COVID-19 pandemic led to nearly universal development of distress and an increased prevalence of depression and generalized anxiety disorder among front-line (FL) healthcare workers (HCWs) [1,2,3]. The prevalence of distress, depression, and generalized anxiety disorder among FL HCWs remained greater than in non-FL HCWs at least 1 year after the pandemic ended [2]. The disaster psychiatry literature strongly recommends interventions to support the mental health (MH) of individuals following major disasters, including screening for psychopathology, linkage to formal psychiatric care for those in need following disaster, and supportive care for distressed individuals [4]; however, the vast majority of healthcare organizations (HCOs) were ill-prepared for the psychosocial sequelae of the pandemic [3]. As a result, efforts to alleviate distress and offer formal psychiatric evaluation were generally inconsistent and inadequate [2].

Yet amidst the chaos of the COVID-19 pandemic, the emerging literature indicates that some HCOs were successful in implementing interventions to support the MH of their FL HCWs. Types of interventions included psychiatric screening and linkage to formal MH care, psychotherapy, psychoeducation (PE), medications, and psychological first aid (PFA), all of which have been found helpful among disaster survivors in the disaster mental health literature. A synthesis of the available data on the effectiveness of MH interventions among FL HCWs is essential to inform an MH plan for FL HCWs in future pandemics. By learning from the COVID-19 pandemic and planning the MH response to future pandemics, HCOs may reduce the MH sequelae among FL HCWs. The objective of this systematic review is to examine interventions used to support the MH of FL HCWs during the COVID-19 pandemic and the reported effects of those interventions.

## 2. Methods

All studies published between 1 January 2020 and 1 March 2026 that reported on MH interventions among FL HCWs during the COVID-19 pandemic were identified using PubMed, PsycINFO, Scopus, EMBASE, Cochrane Database of Systematic Reviews, Google Scholar, Medline, and Web of Science. Studies that included MH interventions outside of the COVID-19 pandemic if at least part of the intervention occurred during the pandemic. Institutional review board review, ethics committee approval, and informed consent were not needed because data were obtained from existing published literature. Computer-based search terms and inclusion and exclusion criteria were based on prior systematic reviews and meta-analyses conducted to evaluate psychological outcomes and interventions among FL HCWs during the pandemic (Table 1 and Table 2) [2]. Studies published in peer-reviewed journals that reported data on interventions to support the MH of FL HCWs were included. Duplicate identical studies from the search were identified by searching duplicate titles within Microsoft Excel and were removed by TNA. Articles identified in the search were screened using the approaches recommended by the Preferred Reporting Items for Systematic Reviews and Meta-Analysis (PRISMA 2020, see Supplementary Materials) by 2 of the study’s authors (TNA and CSN) [5,6]. Disagreements were resolved by discussion between the reviewers until consensus was reached.

Two of this study’s authors (RMR and HJP) independently assessed the full-text articles included after review of the title and abstract and extracted the following information from each article into a systematic form for recording details of intervention design, country, intervention period, sample size, and intervention outcomes. Disagreements were resolved by discussion between the reviewers until consensus was reached. Multiple studies of the same intervention within the same population and repeated identical publications were not duplicated in the review; if the same intervention within the same population was described in >1 published manuscript, the manuscript with a superior randomized design was preferentially chosen. Studies of the same intervention were identified by searching duplicate authors within Microsoft Excel and examining all references by an author (done by RMR and HJP). Included studies were then categorized based on the primary intervention type using a disaster MH framework. Categories of interventions included medications, therapy, psychoeducation (PE) and/or psychological first aid (PFA), screening measures for psychiatric disorders, mindfulness, and multi-modal interventions.

### Statistical Analysis

The quality of non-randomized studies was assessed by the modified version of the Newcastle–Ottawa Scale (NOS) [5]. Studies with 7–9 stars were deemed good quality, 4–6 stars fair quality, and 0–3 stars poor quality [5]. Risk-of-bias assessment for randomized studies was assessed through a revised Cochrane risk-of-bias tool for randomized trials (RoB 2.0).

A narrative synthesis was planned based on intervention type: identification of psychopathology and linkage to care, PE/PFA, psychotherapy, mindfulness programs, medications, and multiple/combined psychosocial support interventions. The study’s reported primary outcome was used to reduce risk of bias. Quantitative data synthesis was not planned due to the marked heterogeneity of outcomes measured. The planned presentation of results included tables with study characteristics and outcomes. Limitations of this method include lack of quantitative assessment.

## 3. Results

**Figure 1:**
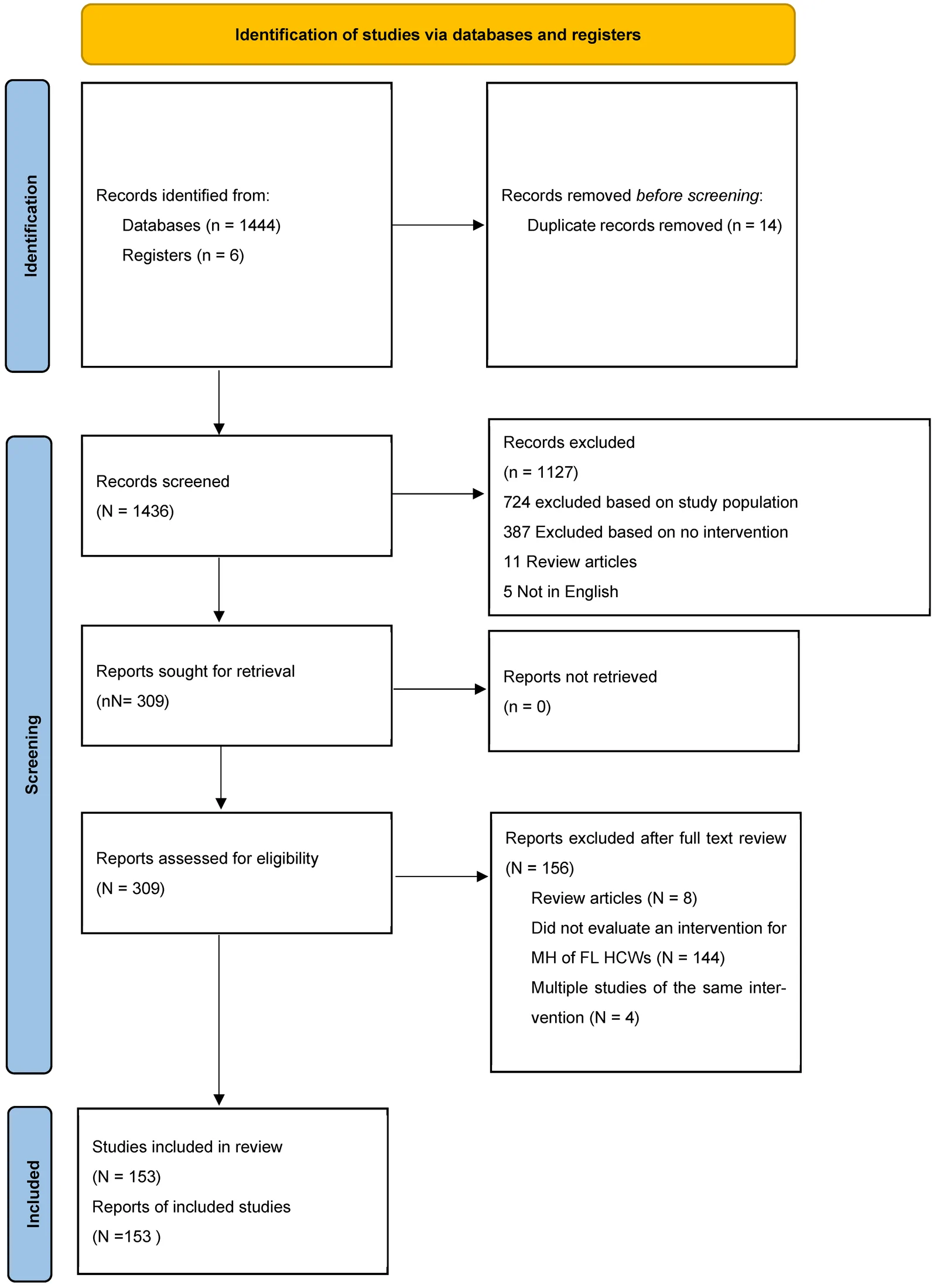
Findings from systematic review.

Searches of PubMed, PsycINFO, Scopus, EMBASE, Cochrane Database of Systematic Reviews, Google Scholar, Medline, and Web of Science were completed on 1 March 2026, generating 1436 records. Of the 1436 records, 1127 were excluded after review of the title and abstract, 309 were reviewed at length, and 153 were included (Figure 1).

Characteristics of included studies are outlined in Table 3. A total of 108 studies were cohort and 45 studies were randomized. Risk of bias judgment was low by RoB 2.0 for all included randomized studies. NOS scores for included non-randomized studies are included in Appendix A.

Full search terms are available in Appendix B. Interventions in these studies included identification of risk for psychopathology and linkage to mental health care (N = 3), PE/PFA (N = 44), psychotherapy (N = 26), mindfulness programs (N = 58), medications (N = 5), and multiple/combined psychosocial support interventions (N = 17). Studies most commonly came from the USA (N = 49) and China (N = 19). Seventy-one percent of participants were female.

Table 4 presents studies of psychological screening and linkage to mental health care. These studies relied on symptom screening tools, and mental health referrals were made for individuals screening positive on the screening tools. Symptom levels were found to be high and many individuals with positive screens were directed to an appropriate level of MH care based on the severity of their symptom scores. In one study, depressive symptoms were severe for 7% of screened participants, anxiety symptoms severe for 14%, psychotic symptoms severe for 2%, and suicidal ideation for 1% [6]. These findings underscore the importance of detection of psychopathology and providing appropriate treatment for it.

Table 5 summarizes studies evaluating PFA, coaching, and psychoeducation (PE) interventions. Of the 44 PE/PFA interventions, 42 (95.5%) achieved the primary outcome measure reported in the study, 1 (2.3%) did not achieve the primary outcome, and 1 (2.3%) did not report results. Primary outcome measures indicated feasibility and acceptability of PE and PFA interventions in a variety of formats: online modules, Zoom meetings, in-person peer support groups, and coaching. Improvements in well-being scores, Generalized Anxiety Disorder-7 (GAD-7), Patient Health Questionnaire-9 (PHQ-9), and Pittsburgh Sleep Quality Index (PSQI) were reported.

Table 6 presents studies evaluating psychotherapies, which included both in-person and online sessions. It is generally agreed that psychotherapies applied in disaster settings need to be circumscribed and crisis-oriented, and can be delivered by trained crisis counselors. Types of therapy included eye movement desensitization and reprocessing (EMDR), cognitive behavioral therapy (CBT), and dialectical behavioral therapy (DBT), as well as biofeedback training for heart rate variability. Group and individual psychotherapy sessions were evaluated. Outcomes were largely favorable, with improvements in Beck Anxiety Inventory (BAI), Beck Depression Inventory (BDI), Symptom Checklist-90 (SCL-90), and PHQ-9 noted in various studies and only 2 studies reporting no change in the primary outcome.

Table 7 lists studies of mindfulness interventions which included programs for yoga, meditation, music therapy, tai chi, and emotional freedom techniques. Interventions were delivered in person, remotely, and via online web applications. Of the 58 mindfulness interventions, 53 (91.3%) included a pre- and post-psychiatric symptom screening survey, and of those, 48 (90.6%) demonstrated improvement in symptoms following the intervention.

Table 8 presents studies of interventions involving multiple or combined types of interventions, which typically included an online PE program, an outreach support center or hotline, and optional psychotherapy, as well as systematic workplace adjustments for the mental well-being of employees. These interventions largely focused on outcomes of feasibility and acceptability with high utilization among FL HCWs. Of the five interventions that included a symptom screen as a primary outcome, four (80%) demonstrated improvement in screening scores after the intervention.

Table 9 shows studies that tested treatments involving medications for FL HCWs, which included psilocybin (N =2), cannabidiol (N = 2), and ketamine-facilitated psychotherapy (N = 1). Each study met its primary outcome of improvement in a psychiatric screening survey, though the surveys utilized to assess outcomes varied. Three of these medication studies evaluated participants with known pre-existing psychopathology and/or positive psychiatric screening surveys at baseline, and the two cannabinoid studies appear to have applied the intervention more broadly.

Odds ratios and Forest plots could not be generated because of substantial variability in outcomes measured for each type of intervention. Examination of this collection of articles revealed numerous methodological limitations. These studies may have had potential for sampling bias, enrolling participants who were especially eager for MH interventions, favoring positive results. For several studies, sample size likely limited statistical power, especially those with <20 (or even just 1) participants. These studies relied extensively on screening tools without full diagnostic evaluation, in which findings pertaining to true psychopathology are limited. The symptom screening and assessment tools were varied and inconsistent, making meaningful comparisons across studies difficult. Fidelity to intervention methods was not always made clear, providing potential threats to the validity of the interventions actually applied. Few studies considered outcomes in relation to pre-existing psychopathology, limiting the ability to attribute outcomes outside of pre-disaster status and to interventions applied. Only some studies utilized adequate comparison or control groups, further limiting the ability to attribute outcomes to the interventions.

## 4. Discussion

This systematic review examined published reports on interventions used to promote the psychological well-being of FL HCWs during the COVID-19 pandemic. The majority of studies found their interventions to be beneficial, largely based on user satisfaction scores and symptom screening scores. Importantly, symptoms in the vast majority of control arms also improved over time, though not to the degree that symptoms in intervention arms improved. This indicates that caution is warranted in studies reporting outcomes not utilizing a control group, as it indicates that mental health can be expected to improve to at least some extent on its own, and that the improvement cannot all necessarily be attributed to the intervention. Clinically, it suggests that distress emerging during FL work, like the distress known to follow other types of major disasters, can be expected to improve over time in most individuals; however, the interventions provided in the reviewed studies to alleviate distress will likely accelerate this improvement.

MH interventions for FL HCWs during the COVID-19 pandemic were divided into categories loosely based on the framework for MH response to disaster outlined in the disaster psychiatry literature. According to this literature, the first step in the MH response to disasters is identification of psychopathology, which for large populations may require initial symptom screening to detect individuals at risk for psychopathology, who will then need formal psychiatric assessment and provision of appropriate care. The disaster literature has documented psychopathology in a substantial proportion of disaster survivors, indicating the need for detection and linkage of psychopathology to mental health services [4]. Three studies in this review implemented symptom screening protocols and linked those at risk to MH care. Given that identifying psychopathology and linkage to appropriate formal evaluation and MH care is the critical first step, it is noteworthy that only three studies focused on this type of response to FL HCWs. Further emphasizing the importance of this first step is that, in line with disaster psychiatry research findings [9,56,63], the studies in the current review found that high levels of anxiety and depressive symptoms were common in FL HCWs, with up to 55% having positive GAD-7 screens [56]. It turned out that psychological screening was embedded in a few of the studies with multiple/combined interventions: 10% of individuals accessed formal MH services [41], and in another study 3.4% required formal psychiatric evaluation after screening by an MH liaison [61]. Importantly, while HCOs may fear liability or lack of receptiveness to MH screening measures, these studies suggest that these procedures are well-received and succeed in identifying many individuals who would benefit from formal psychiatric evaluation and linkage to appropriate care.

A second step in MH response to disasters, according to the disaster psychiatry literature, is to offer supportive care to distressed individuals [4]. Distress is any negative emotional or physical response to a stressor and is nearly universal and hence normative following disaster [1,2,4]. It then follows that supportive interventions may be broadly helpful for most members of affected groups. Examples of supportive interventions used in the reviewed studies of FL HCWs include PE, PFA, brief crisis-focused psychotherapy, and mindfulness activities such as yoga or meditation. The studies in this review generally agreed that these types of interventions were helpful to reduce symptoms of anxiety and depression in FL HCWs during the COVID-19 pandemic and that in a future pandemic, implementation of MH screening coupled with expedited access to psychiatry services for FL HCWs is warranted—which brings us back to the critical first step in disaster psychiatry response provided in the existing literature.

Psychotherapy interventions included EMDR, CBT, and DBT in individual or group settings and with both in-person and telehealth options. Each type of therapy demonstrated a reduction in depressive and/or anxiety symptoms in the studies reviewed. This benefit seemed to be maintained in both the individual and group settings as well as both in-person and remotely. These findings suggest that in future pandemics, offering free and confidential psychotherapy services either in person or remotely can help to alleviate depressive and anxiety symptoms among FL HCWs. An important caveat, however, is that individuals determined to have serious psychopathology risk need to receive formal psychiatric evaluation and care as their first step.

The findings of this review also demonstrated considerable apparent benefit of PE and PFA for FL HCWs. PE consists of teaching individuals about the warning signs of psychopathology, normalizing feelings of distress, and teaching self-care techniques. PFA is an intervention applied in the aftermath of acute disaster designed to comfort and support individuals in disaster and often includes PE. PFA was derived from expert consensus and is not evidence-based, but the studies in this review provide evidence that PFA appears safe and effective for this population. The positive findings reported in these studies suggest that provision of PFA via social workers, psychologists, coaches, or trained peer supporters can be beneficial in future pandemics; further, PE administered via a mobile application, website, or in-person can be implemented to help alleviate depressive and anxiety symptoms in FL HCWs in these high-demand situations.

Mindfulness interventions that support distressed individuals also were reported by these studies to show benefit for depressive and anxiety symptoms among FL HCWs. Mindfulness interventions can include yoga, relaxation therapy, music therapy, meditation, breathing exercises, and progressive muscle relaxation, among myriad others. The breadth of mindfulness activities reported in the literature suggests that a plethora of potentially available mindfulness options may be helpful and that institutions can choose their modalities based on the preferences of FL HCWs or may consider employing multiple mindfulness techniques to improve utilization and flexibility.

Returning again to the critical first step of identifying psychopathology and providing appropriate formal treatment based on formal psychiatric evaluation, two matters deserve emphasis. First, symptom screening tools are not diagnostic measures, and positive screens warrant follow-up with full psychiatric assessment for psychiatric illness. Second, pharmacotherapy and evidence-based therapies are the mainstay of psychiatric treatment, both in general practice as well as in the throes of disaster [159]. The relative paucity of medication studies found in this review likely reflects the FL HCW setting of these studies, which is distinct from formal psychiatric treatment settings where medications and psychotherapies are appropriately used. Established medications and psychotherapies for generalized anxiety and depressive disorders among FL HCWs affected by the COVID-19 pandemic have not yet been evaluated, and future studies will be needed to determine their utility, determine specific indications, and demonstrate the most effective means of providing these treatments in this setting. Of note, a few studies reported the use of alternative medications including cannabinoids, ketamine, and psilocybin. These are not mainline psychotherapeutic agents in psychiatric practice, and studies of them captured in this review likely represent efforts to apply cutting-edge tools that are not established as routine applications in disaster-affected populations. The paucity of medication studies in this review suggests that more research is needed to evaluate the effectiveness of standard treatments for depressive and anxiety disorders in this population.

The Section 3 of this review article identified a host of methodological limitations that may hinder attribution of the outcomes to the interventions applied. The quality of included non-randomized studies was poor or fair, with no high-quality studies found. Additional future research will be needed to overcome potential limitations that include sampling bias, small samples, use of screening tools without full diagnostic evaluation, varied symptom screening and assessment tools, fidelity to intervention methods, consideration of outcomes in relation to pre-existing psychiatric status, lack of measured MH outcomes in some studies, and need for adequate comparison or control groups. Future investigations can overcome these limitations, namely through conducting methodologically more robust studies, with representative samples, standardized assessment instruments, and appropriate comparison groups.

It is acknowledged that MH research is notoriously challenging in the throes of disaster, but prior research on disaster MH interventions in other populations indicates that such methodological considerations can be addressed even in disaster settings.

In the meantime, conclusions about the effectiveness of MH interventions used for FL HCWs in disaster settings will need to be tempered with such caveats. Regardless, the extent of positive outcomes of interventions tried and measured by the studies of this review warrants optimism that MH sequelae of pandemics for FL HCWs can be effectively addressed.

Strengths of the systematic review include the broad search with over 1400 results. While prior systematic reviews have been limited to a single type of MH intervention such as mindfulness, this review includes all MH interventions organized into a disaster MH framework. Limitations of the study include the potential for missed relevant studies based on the search terms and inability to conduct statistical analysis on the included studies due to heterogeneity of outcomes. Further, a major limitation of this review is that it is limited to published studies, which place an emphasis on individual efforts to combat distress [i.e., resilience training] and external interventions with insufficient attention paid to system-wide interventions that support employees and reduce their burdens. Few studies in this review outline policies such as maintaining a broad pool of workers, parental leave policies, and adequate time off, likely because these interventions are more resource-intensive and challenging to study; however, systemic interventions may be more important to the MH of FL HCWs than individual interventions. While data on systemic interventions for FL HCWs is very limited, both the disaster MH literature and studies of HCWs during normal operations demonstrate a reduction in distress following implementation of these systemic efforts [160,161,162].

## 5. Conclusions

In conclusion, this systematic review suggests that an established disaster MH response framework may be useful in directing the design of MH responses to FL HCWs in future pandemics. Although many HCOs did not take any action to promote the MH of FL HCWs, the weight of the evidence from the many studies in this review suggests that inaction is unacceptable and results in a high prevalence of unaddressed psychopathology and distress. Planning for the MH of FL HCWs in the next pandemic can begin now within the framework of the disaster mental health literature, offering screening for psychopathology and connection with formal psychiatric services for full evaluation and provision of MH care, as well as supportive care with mindfulness interventions, crisis-oriented counseling, PE, and PFA. Ongoing research of these interventions among FL HCWs with standardized assessment tools and control groups will allow for more thorough comparisons between interventions and a defined set of best practices for supporting the MH of FL HCWs.

## Figures and Tables

**Table 1:** Search terms.

Mental Health	Intervention	Frontline Healthcare Workers	COVID-19
Stress Distress Burnout Compassion fatigue Moral injury Moral distress Trauma Traumatic Post-traumatic stress disorder PTSD Anxiety Anxious Depress Depression Depressive disorder MDD Mental health Psychological Psychopathology Psychiatric OR/#1–#20	22. Intervention 23. Psychological first aid 24. Program 25. Treatment 26. Implementation 27. Diagnostic assessment 28. Therapy 29. Psychotherapy 30. Counseling 31. Psychoeducation 32. Hotline 33. OR/#22–32	34. Doctor 35. Physician 36. Nurse 37. Health personnel 38. Healthcare provider 39. Healthcare worker 40. Frontline 41. Healthcare staff 42. Medical staff 43. Medical worker 44. OR/#34–#43	45. SARS-CoV-2 46. COVID-19 47. COVID 48. 2019 novel coronavirus infection 49. 2019-nCoV 50. Coronavirus 51. OR/#45–50

**Table 2:** Inclusion and exclusion criteria.

Inclusion	Exclusion
Published in English	All languages other than English
Data collected during the COVID-19 pandemic	Data collected only before or after the COVID-19 pandemic
All or part of the analyses targeted an intervention for the MH of FL HCWs caring for patients with COVID-19: physicians, nurses, and advanced practice providers	Reports limited to the context of another pandemic (e.g., MERS, influenza) or event (e.g., earthquake or another natural event) not related to the COVID-19 pandemic
Reporting quantitative or qualitative data regarding an intervention for the MH of FL HCWs during the COVID-19 pandemic	Reports limited to interventions targeting HCWs who did not perform hospital-based work as a member of the primary healthcare team on hospitalized COVID-19 patients (i.e., dentists, orthopedic surgeons)
	Reports limited to interventions targeting other professionals not working in the health system (i.e., police officers, firefighters, teachers) or healthcare students
	Reports on FL HCW MH without an intervention
	Reporting quantitative or qualitative data limited to description of an intervention for the MH of FL HCWs after the COVID-19 pandemic

List of abbreviations: MH = mental health; FL = front line; HCW = healthcare worker; MERS = Middle East respiratory syndrome.

**Table 3:** Study characteristics.

Reference	Intervention Category *	Country	Intervention Year	Intervention Duration (Weeks)	N Participants	Age, Mean (SD) Years or Most Common Age Group	Men, N (%)	Primary Outcome	Primary Outcome Achieved
Back AL et al. [7]	Medication	USA	2022	4	30	38	15 (50)	Change in MADRS	Yes
Gao Q et al. [8]	Multiple	China	2023	4	101	30	37 (37)	DASS-21 at 28 days	No
Rizzi D et al. 2022 [9]	Screening	Italy	2020	24	107	40	44 (41)	Linkage to psychiatric care	Yes
Rolling J et al. [10]	Multiple	France	2020	NR	NR	NR	NR	Relaxation room utilization	Yes
Zingela Z et al. [11]	Therapy	South Africa	NR	20	761	NR	NR	Perceived utility of intervention	Yes
Cheng P et al. [12]	PFA/PE	China	2020	NR	300	NR	NR	NR	NR
Robles R et al. [13]	PFA/PE	Mexico	2020	NR	51	36.4	17 (33)	CGI	Yes
Zhan J et al. [14]	Mindfulness	China	2021	2	98	40	16 (17)	BAI, PSQI	Yes
He C et al. [15]	Multiple	China	2020	4	62	33.37	3 (4.8)	GAD-7, PHQ-9	Yes
Pratt EH et al. [16]	Mindfulness	USA	2021–22	4	102	26.5	6 (6)	Feasibility	Yes
Li JM et al. [17]	Mindfulness	China	2020	2	87	NR	40 (46)	PHQ-9,GAD-7, PSS, AIS	No
Lee D et al. [18]	Therapy	USA	2020–21	1.5	47	NR	14 (29.8)	DASS-21	yes
Bernstein CA et al. [19]	Multiple	USA	2020	NR	NR	NR	NR	Feasibility	Yes
Lewis BR et al. [20]	Medication	USA	2022–24	8	12	40	6 (50)	QIDS-SR-16	Yes
Arts-de Jong M et al. [21]	Mindfulness	Netherlands	2020–22	8	201	40.6	19 (9.5)	PHQ-SADS	No
AlQarni AM et al. [22]	Mindfulness	Saudi Arabia	2020	2	125	33	33 (25.6)	WHO-5, CD-RISC-10, STAI	Yes
Malik M et al. [23]	PFA/PE	USA	2020	NR	1319	NR	NR	Feasibility and acceptability	NA
Blake H et al. PMID 32357424 [24]	PFA/PE	UK	2020	NR	NR	NR	NR	User satisfaction	Yes
Trottier K et al. [25]	PFA/PE	Canada	2021	8	21	39	1 (4.8)	PHQ-9, GAD-7, PCL-5	Yes
Costello Z et al. [26]	PFA/PE	USA	NR	8	107	NR	NR	Feasibility	Yes
Captari LE et al. [27]	Therapy	USA	2020–2021	10	77	49.32	16 (21)	Quantitative (NR) and qualitative measures	Yes
Souza JDS et al. [28]	Medication	Brazil	2020	4	300	34.45	21 (7)	GAD-7 and PHQ-9	Yes
LeGoff DB et al. [29]	Therapy	USA	2020–21	10	103	46	31 (30)	Paid time off quantity	Yes
Thum CC et al. [30]	PFA/PE	Malaysia	2020	NR	NR	NR	NR	NR	NA
Garcia-Vazquez B et al. [31]	PFA/PE	Spain	2023–24	NR	17	43	3 (17.6)	Acceptability and feasibility	Yes
Misra P et al. [32]	other—yoga	India	NR	12	89	37.1	53 (59.6)	DASS-21 and WHO-5	Yes
Torricelli L et al. [33]	Therapy	Italy	2020	4	20	48.5	4 (20)	Qualitative	NR
Ghazanfarpour M et al. [34]	Therapy	Iran	2020	1	95	NR	80 (84.2)	HADS	Yes
O’Keefe VM et al. [35]	PFA/PE	USA	NR	4	56	NR	6 (11)	PROMIS	Yes
Dyer NL et al. [36]	Mindfulness	UK	2020–21	<1	40	43.9	1 (2.5)	Stress and well-being	Yes
Feinstein RE et al. [37]	PFA/PE	USA	2020	NR	NR	NR	NR	NR	NR
Gayed A et al. [38]	PFA/PE	Australia	2020–2021	20	39	NR	11 (28)	DASS-21	Yes
Smith-MacDonald L et al. [39]	Therapy	Canada	NR	7	8	37	0 (0)	Feasibility	Yes
Reyes AT et al. [40]	Mindfulness	USA	2021–22	6	60	35.4	20 (33)	PCL-5, AAQ-II, RRS, MAAS	Yes
Agarwal AK [41]	Multiple	USA	2020–2023	NR	NR	NR	NR	Utilization	Yes
Zhang D et al. [42]	PFA/PE	China	2020	4	60	NR	16 (26.7)	Self-rating anxiety and depression	Yes
Robison R et al. [43]	Medication	USA	NR	6	10	37	5 (50)	NR	Yes
Luo LX et al. [44]	Mindfulness	China	2021	8	NR	NR	22 (NR)	MBI-GS, SCL-70	Yes
Dumarkaite A et al. [45]	PFA/PE	Lithuania	2021	6	168	40.39	5 (1.8)	PHQ-4	Yes
McLean CP et al. [46]	PFA/PE	USA	NR	8	94	NR	17 (18)	Three high-loading single items from the Acceptability of Intervention Measure, Intervention Appropriateness Measure, and Feasibility of Intervention Measure	Yes
Farrel D et al. [47]	Therapy	UK	NR	1	95	46.0 (10.7)	18 (18.9)	ITQ	No
Sanford J et al. [48]	Multiple	USA	NR	NR	NR	NR	NR	NR	NR
Yurtsever A [49]	Therapy	Turkey	2020	4	154	36.3	23 (14.9)	IES	Yes
Kanstrup M et al. [50]	Therapy	Sweden	NR	5	144	41	25 (17.4)	PCL-5	Yes
Hu Y et al. [51]	Mindfulness	China	2020	12	60	30.25	NR	Depressive and anxiety symptoms—survey NR	Yes
Pihlgren SA et al. [52]	Therapy	Sweden	2022	NR	7	40	2 (28.5)	Qualitative	Yes
McCann B et al. [53]	Therapy	Scotland	NR	NR	NR	38.5	NR	Acceptability	Yes
Viswanathan R et al. [54]	Therapy	USA	2020	NR	197	NR	NR	NR	NR
Lefèvre H et al. [55]	Mindfulness	France	2020	4	379	NR	44 (11.6)	NR	NR
Zhang N et al. [56]	Screening	China	2020	4	200	NR	92 (46)	PHQ-9, GAD-7	Yes
Kubickova V et al. [57]	Therapy	UK	2020	4	12	32.92	4 (33.3)	IES	Yes
Bazan GN et al. [58]	Mindfulness	USA		6	52	NR	8 (15.3)	MBI	Yes
Sarwal R et al. [59]	Mindfulness	India	2020	4	150	34.9	132 (88)	MBI	Yes
Sağaltıcı E et al. [60]	Therapy	Turkey	2020	NR	14	34.14	3 (21.4)	BAI, IES	Yes
Geoffroy PA et al. [6]	PFA/PE	France	2020	NR	NR	32	NR (14)	Call duration and quantity	Yes
Gray M et al. [61]	Multiple	USA	2020	NR	1090	NR	NR	Qualitative	Yes
Bureau R et al. [62]	Therapy	France	2020	1	10	37.8	1 (10)	Feasibility	Yes
Cole CL et al. [63]	Screening	UK	2020	NR	NR	NR	NR	NR	NR
Rizzi D et al., 2025 [64]	Therapy	Italy	2021–22	24	225	44	95 (42.2)	NSESSS	No
Azizoddin DR et al. [65]	Mindfulness	USA	NR	12	32	NR	13 (40.6)	PHQ-9, GAD-7	Yes
Wang CC et al. [66]	Multiple	USA	NR	8	1	NR	NR	QIDS-SR-16, GAD-7, CAPS-5	Yes
Appelbom S et al. [67]	Multiple	Sweden	2020	NR	NR	NR	NR	Feasibility	Yes
Bigman-Peer N et al. [68]	Mindfulness	Israel	2022	<1	86	36	17 (19.8)	MBI, GAD-7	Yes
Gupta S et al. [69]	Therapy	India	2020	4	35	NR	NR	DASS-21, IES	No
Fung K et al. [70]	PFA/PE	Canada	2021–2022	6	325	NR	NR	NR	NR
Wild J et al. [71]	Therapy	England and Scotland	2020–22	6	103	38.73	29 (28.2)	NR	Yes
Ache ALDS et al. [72]	PFA/PE	Brazil	2020–2021	4	709	37.7	88 (12.4)	Symptom improvement	Yes
Kameno Y et al. [73]	Therapy	Japan	2020	12	31	28.3	2 (6.5)	Kessler’s psychological distress	Yes
Azizi TH et al. [74]	PFA/PE	Iran	2023	6	77	31	18 (23.3)	Kessler’s psychological distress and CDRS	Yes
Bonetto C et al. [75]	Mindfulness	Italy	NR	7	33	NR	7 (21.2)	PHQ-9, GAD-7, IES	No
Beverly E et al. [76]	Mindfulness	USA	2021	<1	102	NR	29 (28.4)	Subjective stress scores	Yes
Blake H et al. PMID 33333913 [77]	PFA/PE	UK	2020	17	819	NR	85 (10.4)	Utrecht Work Engagement Scale	Yes
Korman MB et al. [78]	Multiple	Canada	2020	78	NR	NR	NR	Qualitative	Yes
Castro Ribeiro T et al. [79]	Therapy	Spain	2021	5	21	37.7	0 (0)	PHQ-9, STAI, PCL-5	Yes
Rodriguez-Vega B et al. [80]	Mindfulness	Spain	2020	NR	150	38.6	30 (20)	Feasibility and acceptability	Yes
DeVylder EK et al. [81]	Multiple	USA	NR	NR	9	38.9	1 (11.1)	Feasibility and acceptability	Yes
Samadi S et al. [82]	Multiple	Iran	2020–2021	NR	NR	NR	NR	NR	NR
Rubin EW et al. [83]	Multiple	USA	2020	NR	71	NR	NR	Feasibility and acceptability	Yes
Gunjiganvi M et al. [84]	Mindfulness	India	2020–2021	2	79	32	40 (50.6)	PHQ-9, GAD-7, ISI	Yes
DiGalbo RT et al. [85]	Mindfulness	USA	2021–2022	8	34	41.3	5 (14.7)	MBI	Yes
Evans LV et al. [86]	Mindfulness	USA	2021	<2	80	35.5	36 (45)	STAI	No
L’Engle K et al. [87]	PFA/PE	USA	2023	6	34	36	3 (8.8)	PHQ-4	No
West K et al. [88]	Mindfulness	USA	2020	<1	20	NR	NR	NR	NR
Connors JN et al. [89]	PFA/PE	USA	NR	8	24	NR	1 (4.2)	PHQ-4	Yes
Osman I et al., 2022 [90]	Mindfulness	Africa	2020	4	55	NR	NR	Qualitative	Yes
Ballesio A et al. [91]	PFA/PE	Italy	NR	NR	NR	NR	NR	NR	NR
Zhou K et al. [92]	PFA/PE	China	2020–2021	6	118	30.4	4 (3.4)	PHQ-9, GAD-7, PSQI	Yes
Kumar N et al. [93]	PFA/PE	Pakistan	2021	12	289	NR	150 (51.9)	Emotional exhaustion (Yongxin and Yimin Model) and occupational calling from Brief Calling Scale	Yes
Gao F et al. [94]	Mindfulness	China	2022–2023	6	54	31	29 (53)	Primary outcome: strength and grip; secondary outcomes: SAS and SDS	Yes
Crippa JAS et al. [95]	Medication	Brazil	2020	4	120	33.6	41 (33.1)	MBI, PHQ-9, GAD-7, PCL-5	Yes
Leland NE et al. [96]	Multiple	USA	2021–2022	NR	94	NR	NR	Feasibility and acceptability	Yes
Vogt KS et al. [97]	PFA/PE	UK	2021–2022	NR	77	39.7	11 (14)	Feasibility and acceptability	Yes
Wei G et al. [98]	PFA/PE	China		NR	176	34 (10)	17 (9.7)	PSQI	Yes
Tran K et al. [99]	Mindfulness	USA	2023	4	36	NR	NR	PSS-10 and WHO-5	Yes
Xu J et al. [100]	Therapy	China	2020	NR	1534	NR	NR	scl-90	Yes
De Kock JH et al. [101]	Mindfulness	UK	2020	8	169	NR	20 (11.8)	PHQ-9, GAD-7	Yes
Gálvez-Herrer M et al. [102]	Therapy	Spain	NR	NR	NR		NR	Feasibility and acceptability	Yes
Romano D et al. [103]	Mindfulness	Canada	NR	NR	NR	NR	NR	Feasibility and acceptability	Yes
Khairallah GM et al. [104]	PFA/PE	Lebanon	2020–2022	104	60	NR	14 (23.3)	Feasibility and acceptability	Yes
Hsieh CW et al. [105]	Mindfulness	Taiwan	2023	8	60	33.15	10 (16.7)	Pandemic fatigue score and resilience score	Yes
Rene R et al. [106]	PFA/PE	USA	2020	17	1324	NR	NR	Feasibility and acceptability	Yes
Wang D et al. [107]	Mindfulness	China	NR	4	602	29	33 (5.5)	OCD, Sleep quality	Yes
Yin Q et al. [108]	Mindfulness	China	2020	8	85	33	17.765	PSS, SCL-90, PSQI	Yes
Kelly F et al. [109]	PFA/PE	South Africa	2020	NR	474	NR	22 (4.6)	Feasibility and acceptability	Yes
Moskowitz JT et al. [110]	PFA/PE	USA	2022	5	554	43.5	60 (10.8)	ACSQ-Me, OLBI	Yes
Monette DL et al. [111]	PFA/PE	USA	2020	<1	68	NR	NR	Feasibility and acceptability	No
Shopen N et al. [112]	PFA/PE	Isreal	2019–2021	<1	13	NR	NR	MBI	Yes
Vagedes J et al. [113]	Biofeedback training	Isreal	2022	<1	114	37.3	41 (36.0)	Heart rate variability	No
Hung CL et al. [114]	Mindfulness	Taiwan	2021	4	26	NR	0 (0)	Heart rate variability indicators, Nurse Stress Checklist, and Copenhagen Burnout Inventory	Yes
Moody KM et al. [115]	PFA/PE	USA	2020–2021	78	NR	NR	NR	Wellness scores	Yes
Kim S et al. [116]	Mindfulness	Canada	2020–2021	26	130	NR	NR	Resilience score	Yes
Zador L et al. [117]	MM	USA	2019–2020	52	54	NR	43 (79.6)	Well-being index	Yes
Ward MC et al. [118]	PFA/PE	Australia	2020–2021	32	55	NR	NR	WHO-5	Yes
Lee SK et al. [119]	Mindfulness	USA			102	39.3	23 (22.6)	Ecological momentary assessments (EMA) completion	Yes
James TT et al. [120]	PFA/PE	USA	2020–2021	52	101	NR	48 (47.5)	MBI	Yes
Castillo-Sánchez G et al. [121]	Mindfulness	Spain	2020	16	359	NR	18 (5.0)	Feasibility and acceptability	Yes
Cheng W et al. [122]	PFA/PE	China	2020	6	156	NR	41 (26.3)	Daily mood scores	Yes
Allsopp K et al. [123]	Screening	England	2021–2022	52	62	NR	NR	Feasibility and acceptability	Yes
Hartung K et al. [124]	PFA/PE	USA	2022	<1	573	NR	NR	American Medical Association organizational biopsy measurement	Yes
Adair KC et al. [125]	Mindfulness	USA	2021–2023	<1	574	NR	57 (10.1)	Emotional recovery and emotional thriving scores	Yes
Kevern T et al. [126]	Therapy	USA	202–2021	<1	91	NR	55 (60.4)	Feasibility and acceptability	Yes
Lu C et al. [127]	PFA/PE	China	2020–2021	<1	62	30	6 (9.7)	IERQ	Yes
Nijland JWHM et al. [128]	Mindfulness	Netherlands	NR	NR	86	42.4	15 (17.4)	PSS-10	Yes
Amsalem D et al. [129]	PFA/PE	USA	NR	NR	350	34.8	84 (24)	Openness to seeking health screening	Yes
Al Ozairi A et al. [130]	Mindfulness	Kuwait	2020–2021	NR	56	NR	10 (17.9)	GAD7	Yes
Cepeda-Lopez AC et al. [131]	Mindfulness	Mexico	2021–2022	8	643	34.8	116 (18.0)	Subjective well-being and resilience	Yes
DeTor NR et al. [132]	Mindfulness	USA	2020	8	554	44.14	61 (11.0)	PHQ-4	No
Kanchibhotla D et al. [133]	Mindfulness	India	2020	8	92	43.1	38 (41.3)	DASS-21, PSQI	Yes
Fiol-DeRoque MA et al. [134]	Mindfulness	Spain	2020	12	482	41.37	81 (16.8)	DASS-21, DTS, ISI	Yes
Gheradrdi-Donado ECDS et al. [135]	Mindfulness	Brazil	2020	8	54	33.8	9 (16.7)	BDI, BAI	Yes
Hsieh HF et al. [136]	Mindfulness	China	202–2021	1	79	37	NR	Smart watch stress levels	Yes
Ibrahim K et al. [137]	Mindfulness	Indonesia	2020	4	50	35.2 (7.9)	9 (18)	WEMWBS	Yes
Keng SL et al. [138]	Mindfulness	Singapore	2021	3	80	30.18	8 (10.0)	DASS-21	Yes
Nestor MS et al. [139]	Mindfulness	USA	2020	10	130	44.25	43 (33.1)	BSI 18, ISI, MBI	Yes
Nourian M et al. [140]	Mindfulness	Iran	2020	NR	NR	NR	NR	PSQI	Yes
Osman I et al. 2021 [141]	Mindfulness	south Africa	2020	4	47	NR	NR	PSS, MAAS, aMBI	Yes
Prado K et al. [142]	Mindfulness	NR	NR	4	100	NR	13 (13)	PSS	Yes
Thimmapuram J et al. [143]	Mindfulness	USA	NR	4	155	46 (11.1)	42 (27.1)	UCLA loneliness score and PSQI	Yes
Vajpeyee et al. [144]	Mindfulness	India	NR	NR	240	NR	175 (83.77%)	DASS-42	Yes
Yildirim D et al. [145]	Mindfulness	Turkey	2020	<1	102	28.33	NR	STAI-I	Yes
Meredith LS et al. [146]	PFA/PE	USA	2020–2021	<1	4154	NR	872 (21.0)	PTSD checklist and Kessler6	Yes
Timmins G et al. [147]	PFA/PE		NR	NR	NR	NR	NR	Binary indicators of agreement with 6 questions about experiences with the SFA intervention	Yes
Dincer B et al. [148]	Mindfulness	Turkey	2020–2021	NR	72	33.45	6 (8.3)	SUD and STAI-I	Yes
Teall AM et al. [149]	PE/PFA	USA	NR	6	188	NR	NR	Feasibility and acceptability	Yes
Sun L et al. [150]	PE/PFA	China	2020	8	66	34.1 (4.9)	0 (0)	SCL-90, Campbell Index of Well-being and Work Stress Reaction Scale	Yes
Maunder RG et al. [151]	Multiple	Canada		NR	538	38.5	85 (15.8)	MBI EE score	Yes
Rosen B et al. [152]	PE/PFA	Canada	2021	NR	24	NR	4 (16.7)	Feasibility and acceptability	Yes
Miyoshi T et al. [153]	Mindfulness	Japan	2021	12	13	49	2 (15.4)	Heart rate and stress cortisol levels	Yes
Chang J et al. [154]	Mindfulness	USA	2021–2022	26	84	NR	42 (50.0)	Feelings of acknowledgment and SPFI	Yes
Giordano F et al. [155]	Mindfulness	Italy	2021	<1	34	31.8	11 (32.4)	MTC total score	No
Sockalingam S et al. [156]	Mindfulness	Canada	2020	NR	426	NR	NR	Feasibility and acceptability	Yes
Othman SY et al. [157]	Mindfulness	Egypt	2021	<1	60	NR	15 (25.0)	MBI EE score	Yes
Iyadurai L et al. [158]	Mindfulness	UK	2021	<1	86	37.9	15 [17.4)	Intrusive thought frequency	Yes

* Interventions were categorized as medications (Medication), psychological first aid/psychoeducation/coaching/peer support (PFA/PE), psychotherapy (Therapy), multi-modality (Multiple), Mindfulness/yoga/other (Mindfulness), psychopathology screening (Screening). Abbreviations: NR: not recorded; MADRS: Montgomery–Asberg Depression Rating Scale; ITQ: International Trauma Questionnaire; PHQ-9: Patient Health Questionnaire-9; GAD-7: Generalized Anxiety Disorder-7; DASS-21: Depression Anxiety Stress Scales; CGIs: Clinical Global Impressions; PSQI: Pittsburgh Sleep Quality Index; BAI: Beck Anxiety Inventory; PSS: Perceived Stress Scale; AIS: Athens Insomnia Scale; QIDS-SR-16: Quick Inventory of Depressive Symptomatology (16 item); PHQ-SADS: Patient Health Questionnaire Somatic, Anxiety, and Depressive Symptoms; WHO-5: World Health Organization-5 well-being index; CD-RISC: Connor–Davidson Resilience Scale; STAI: State Trait Anxiety Inventory; PCL-5: PTSD Checklist for DSM-5; HADS: Hospital Anxiety and Depression Scale; PROMIS: Patient-Reported Outcomes Measurement Information System; AAQ-II: Acceptance and Action Questionnaire; RRS: Ruminative Responses Scale, MAAS: Mindful Attention Awareness Scale; MBI-GS: The Maslach Burnout Inventory-General Survey; SCL-70: Symptom Checklist; ITQ: International Trauma Questionnaire; IES; MBI: Maslach Burnout Inventory; NSESS: National Stressful Events Survey; CAPS-5: Clinician-Administered PTSD Scale for DSM5; ISI: Insomnia Severity Index.

**Table 4:** Identification of risk for psychopathology and linkage to mental health care.

Reference	Subjects	Description of Intervention	Control Group Y/N	Results
Rizzi D et al., 2022 [9]	FL HCWs	Psychologist-administered psychopathology screening and linkage to care for positive screens.	No	Following screening, 280 individual support sessions were offered. PTSD symptoms were reported as severe by 13% of the FL HCWs. Depressive symptoms were determined to be severe for 7% and anxiety symptoms severe for 14%. Severe psychotic symptoms were experienced by 2% and severe suicidal thoughts by 1% of the FL HCWs.
Zhang N et al. [56]	FL HCWs	HCWs with mild to moderate symptoms on GAD-7 and PHQ-9 received group psychological intervention and PE; those with severe symptoms had individual therapy, psychiatric clinics, medication administration, and time off work.	Yes (non FL HCWs)	No difference in PHQ-9; GAD-7 scores lower after intervention (*p* < 0.05). Positive GAD-7 in 55.7%.
Cole CL et al. [63]	FL HCWs	Phase 1—screening and PFA; Phase 2—CBT-based interventions; Phase 3—a screen and treat approach.	No	NR

Abbreviations: PHQ-9: Patient Health Questionnaire-9; GAD-7: Generalized Anxiety Disorder-7; PE: psychoeducation; PFA: psychological first aid; CBT: cognitive behavioral therapy; NR: not reported.

**Table 5:** Studies of PFA, coaching, and psychoeducation [PE].

Reference	Study Population	Description of Intervention	Control Group Y/N [Treatment in Control Arm]	Results
Cheng P et al. [12]	FL HCWs	Individual PE and group PE and therapy via WeChat app	No	NR
Robles R et al. [13]	FL HCWs	Free 24 h hotline providing PFA, link to resources	No	Decrease in CGI severity: initial 7.3 (CI 2.1) vs. 2.4 (CI 2.4) at end of intervention, *p* < 0.001
Malik M et al. [30]	FL HCWs	Wellness rounds providing PFA	No	NR; 20% FL HCWs in wellness rounds were in acute crisis
Blake H et al. PMID 32357424 [31]	FL HCWs	Digital learning package implementing PE and PFA	No	High user satisfaction with content, usability and utility
Trottier K et al. [32]	FL HCWs	RESTORE (Recovering from Extreme Stressors Through Online Resources and E-health)	No	Improved PHQ-9 and GAD-7 scores at end of intervention compared to beginning
Costello Z et al. [33]	Security guards in COVID-19 wards	Resilience and mental health awareness workshops	No	Qualitative feedback indicated a generally positive reception
Thum CC et al. [37]	FL HCWs	Virtual PFA	No	NR
Garcia-Vazquez B et al. [38]	FL HCWs	“Doing What Matters in Times of Stress” (DWM), a web-based, self-help tool offered in hybrid and remote formats	No	Participation rate was 44% and retention rates were 80 and 100% for the hybrid and remote formats. Intervention was perceived as acceptable, appropriate, and feasible
O’Keefe VM et al. [42]	FL HCWs in American Indian/Alaska Native communities	4. Online PFA Training	No	Significant increases in PFA knowledge test scores for 3 of 4 training modules; no change in MH outcomes between baseline and 1 week post-training; positive changes in mental health, burnout, and social well-being between baseline and 3 months post-training
Feinstein RE et al. [44]	FL HCWs	Free 24/7 Healthcare Worker Mental Health COVID-19 Hotline	No	NR
Gayed A et al. [45]	FL physicians	Individual-focused 6-session virtual coaching program	No	DASS-21 scores significantly improved post-intervention (7.44 (95% CI 2.93–11.95), *p* < 0.001) and maintained at 3 months (8.24 (95% CI 3.57–12.91), *p* < 0.001))
Zhang D et al. [48]	FL HCWs	4 h of resourcefulness training	Yes (PE and counseling)	Resourcefulness, resilience, and positive response in the intervention group were significantly higher than control group during the same period (all *p* < 0.05)
Dumarkaite A et al. [51]	FL nurses	Therapist-guided 6-week CBT-based internet-delivered stress recovery intervention	Yes (no intervention)	Intervention improved stress recovery, stress (*d* = −0.49 (−80; −0.18)), anxiety symptoms (*d* = −0.31 (−0.62; −0.01)), depression symptoms (*d* = −0.49 (−0.80; −0.18)) and increasing psychological well-being (*d* = 0.53 (0.23; 0.84))
McLean CP et al. [52]	FL HCWs at the Veterans Health Administration	Brief, multi-session, didactic program providing stress first aid	No	Most participants reported that they “Agree” or “Completely Agree” that the program is appealing to them (86.6%), suitable for VA (93.5%), and doable (90.5%)
Geoffroy PA et al. [6]	All HCWs including FL	24/7 crisis hotline	No	149 calls with a mean of 5.73 calls/day (SD = 3.22). Average call duration was 18.5 min (min = 1; max = 65 min; SD = 14.7). 105 (70.5%) were referred for additional support
Fung K et al. [70]	FL HCWs	PACER program consisting of 6, 1 h weekly, online self-guided PE modules and weekly, 90 min facilitated online group videoconferencing sessions.	No	NR
Ache ALDS et al. [72]	FL HCWs	60 min PE session, followed by distribution of videos over 4 weeks	No	558 participants (78.7%) reported improved symptoms after 1 month
Azizi TH et al. [74]	FL nurses	6 weekly online educational sessions	Yes (no intervention)	Increase in mean resilience score after intervention and at 6-month follow-up compared to the control group (*p* < 0.001)
Blake H et al. PMID 33333913 [77]	FL HCWs	Wellness centers with space for relaxation and 2 workers trained in PFA	Yes (no intervention)	Well-being was higher in those who accessed a well-being center; no significant differences in perceived job stressfulness, job satisfaction, presenteeism or turnover intentions
L’Engle K et al. [87]	FL HCWs in Federally qualified health center	6 weekly 20 min coaching sessions; daily messages with PE content	No	Stress (z = −2.310, *p* = 0.021) and sleep difficulties (z = −2.706, *p* = 0.007) significantly decreased at follow-up; no change in anxiety or depression symptoms
Connors JN et al. [89]	Emergency Medicine physicians with self-reported MH symptoms	Virtual peer support intervention	No	Modest improvement in 9 of 12 symptom measurements with marginal significance (*p* < 0.10) for improvement in guilt (20, Effect Size (ES) = 0.45) and depression (21, ES = 39). Qualitative findings revealed high overall benefit.
Ballesio A et al. [91]	FL HCWs	PE for sleep problems	No	NR
Zhou K et al. [92]	FL HCWs	6 weeks, with structured e-aid CBT-I animation courses every day	Yes (no intervention)	Compared with subjects in the control group (7.1 ± 5.6, 7.3 ± 5.1), subjects in the eCBT-I group (3.7 ± 3.4, 4.2 ± 4.1) had lower scores on the GAD-7 and PHQ-9 scales after treatment (*p* < 0.05)
Kumar N et al. [93]	FL HCWs	3-month program on responsible leadership practices including communication and MH support	No	Occupational calling increased from 5.162 (SD = 1.151; *t* = 76.253) to 5.403 (SD = 1.054; *t* = 87.138; *p* < 0.001). Emotional exhaustion decreased from 4.386 (SD = 1.037; *t* = 71.879) to 3.614 (SD = 1.336; *t* = 45.987; *p* < 0.001)
Vogt KS et al. [97]	FL ICU nurses	Resilience boosting coaching program delivered remotely at 5 time points	No	PHQ and burnout scores improved from session 1 to 3, not with subsequent sessions
Wei G et al. [98]	FL HCWs	Intervention based on self-transcendence theory [spiritual activity to extend upper limits of goals and existing values with meaning of truth, value, and love of life]	Yes (none)	Significant decrease in PSQI score after intervention compared to control group
Rene R et al. [106]	FL HCWs	Psychoeducation on sleep, nutrition, grief, anxiety, and yoga	No	388 sessions were offered with 1324 participants; 92% of participants felt the sessions helped them
Kelly F et al. [109]	FL HCWs	5-part module on various mental health topics	No	Participants exhibited significant increases in knowledge, confidence, resilience-building behavior, resilience, and well-being scores
Moskowitz JT et al. [110]	FL HCWs	PARK: possible affect regulation skills; a five-week, online, self-guided coping skills intervention	Yes (none)	No statistically significant difference between intervention and control groups in ACSQ-Me, OLBI
Monette DL et al. [111]	FL HCWs in emergency department	One-hour weekly debriefing sessions focused on empathy and normalizing reactions	No	Emergency clinicians found these sessions to be useful (78.8+/−17.6); interquartile range: 73–89]
Shopen N et al. [112]	FL HCWs	PE workshop	No	Intervention had no significant impact on burnout levels (*p* < 0.41, Friedman test]
Moody KM et al. [115]	FL HCWs	Weekly Webex with mindfulness, nutrition, game, DEI	No	Mean change in distress level was −1.67 (SD, 2.02), and 69.4% of participants (95% CI, 62.9–75.9%) experienced decreased distress; higher number of sessions attended associated with higher mean changes
Ward MC et al. [118]	FL physician trainees	Peer-to-peer support, workshops, and online didactics	No	WHO-5 increased by 14.9 (95% CI 0.5 to 29.3), *p* = 0.006; mean reduction in CBI of 6.8 points (95% CI: −14.4 to 0.7)
James TT et al. [120]	FL HCWs	3 hospitalists designated as wellness warriors—coping with vaccine, case debriefs, ICU updates	Yes (none)	Post-intervention, intervention group reported 32% burnout and controls reported 56% (*p* = 0.024). High wellness support was reported by 48% of the intervention group vs. 0% of controls (< 0.001)
Cheng W et al. [122]	FL HCWs	5 PE modules	No	Daily mood score ranged from 7 to 9 out of 10 and was considered positive
Hartung K et al. [124]	FL HCWs	Toolkit with sessions about leadership, engagement, well-being; gratitude rounds	No	Burnout on AMA score 51 to 35% (*p* < 0.0001)
Lu C et al. [127]	FL nurses	Confiding about work-related issues for at least 5 min twice over 8 weeks.	Yes (none)	IERQ scores showed the effect of the intervention was significant, *F* (1, 62) = 6.981, *p* = 0.010
Amsalem D et al. [129]	FL HCWs	Short video of nurse describing how she knew and results of seeking MH support [2 groups, control none] 14 days later [one group gets booster]	Yes (1. no booster or 2. no intervention)	Use: (video + booster group: from 7.9 (95% CI 7.3–8.4) to 9.2 (95% CI 8.7–9.7), *p* < 0.001, Cohen’s *d* = 0.50; video group: from 8.3 (95% CI 7.9–8.8) to 9.4 (95% CI 9.0–9.7), *p*< 0.001, Cohen’s *d* = 0.46). Baseline to 30-day follow-up video + booster group: from 7.9 (95% CI 7.3–8.4) to 9.7 (95% CI 9.3–10.1), *p* < 0.001
Al Ozairi A et al. [130]	FL HCWs	Online group sessions with psychologist and psychiatrist providing PE	No	Significant improvement in GAD-7 score −4.4 (*p* < 0.001]
Meredith LS et al. [146]	FL HCWs	Site champions reviewed 4 h of training videos and then participated in a 2 h virtual, live peer-to-peer support training delivered by the developer of the adapted intervention	Yes	In HCWs 30 years or younger: a more than 4-point reduction for psychological distress (−4.552 (95% CI, −8.067 to −1.037)) and a nearly 7-point reduction for PTSD symptom scores (−6.771 (95% CI, −13.224 to −0.318))
Timmins G et al. [147]	FL HCWs	PFA	No	Between 48.2 and 59.4% of HCWs agreed or strongly agreed that they: found SFA helpful (48.2%), felt comfortable supporting colleagues (59.4%), would recommend SFA (51.2%), and would continue to use SFA principles (57.2%)
Teall AM et al. [149]	FL nurses	Wellness support sessions with an advanced practice nursing student were approximately 45 min in length; wellness partners were encouraged to meet every 7 to 10 days, with a minimum of 4 coaching sessions to be completed over 6 weeks	No	97.3% shared that the Wellness Partner Program helped them engage in self-care and wellness during the program, and 94.7% agreed or strongly agreed that the initiative helped them improve their mental and physical health
Sun L et al. [150]	FL nurses	Balint group intervention and group time management training once weekly for 8 weeks	Yes	Statistically significant improvements in SCL-90 and total well-being score after 8 weeks in intervention group but not control group
Rosen B et al. [152]	FL HCWs	Sessions led by certified coaches	No	Resilience Coaching offered opportunities for connection, encouragement to attend to personal wellness, and coping skills and facilitated HCWs in accessing clinical mental health support when requested

Abbreviations: NR: not reported; FL HCW: front-line healthcare worker; MADRS: Montgomery–Asberg Depression Rating Scale; MSBR: mindfulness-based stress reduction; PHQ-9: Patient Health Questionnaire-9; GAD-7: Generalized Anxiety Disorder-7; QIDS-SR-16: Quick Inventory of Depressive Symptomatology [16 item]; PCL-5: PTSD Checklist for DSM-5; PE: psychoeducation; NR: not recorded; CGI: Clinical Global Impressions; PFA: psychological first aid; DASS-21: Depression Anxiety Stress Scales; SD: standard deviation; PACER: Pandemic Acceptance and Commitment to Empowerment Response; SSI-EP: Single-Session Intervention with Enhanced Psychoeducation; MH: mental health; CBT-I: cognitive behavioral therapy for insomnia.

**Table 6:** Psychotherapy studies.

Reference	Study Population	Description of Intervention	Control Group Y/N [Treatment in Control Arm]	Results
Zingela Z et al. [18]	FL HCWs	Group counseling session	No	90.5% found it informative and useful for managing stress
Lee D et al. [25]	FL HCWs	Heart rate variability biofeedback training in 30 min guided sessions every other day via telehealth	Yes (in-person heart rate variability biofeedback)	Depression, anxiety, and stress scores decreased in control and intervention group; no significant differences between groups
Captari LE et al. [34]	FL chaplains	Spiritually integrated support group led by psychotherapist	No	Significant decreases in burnout and spiritual/moral struggles from pre- to post-intervention and increases in sense of resilience and flourishing
LeGoff DB et al. [36]	FL HCWs with delayed recovery from COVID-19 infection	3 sessions of work-focused CBT	No	Duration of recovery and return to work were reduced along with improvements in work-relevant (40%) and adaptive functioning (31%)
Torricelli L et al. [40]	FL HCWs	EMDR-Integrative Group Treatment Protocol	No	Participants appreciated the group as a safe space, despite individual differences
Ghazanfarpour M et al. [41]	FL HCWs	7 telehealth counseling sessions over 7 days	Yes (no intervention)	Anxiety related to coronavirus and to perceived risk of illness were significantly lower in the intervention group in comparison with the control group (*p* = 0.001)
Smith-MacDonald L et al. [46]	FL HCWs	Evidence-informed online Acceptance and Commitment Therapy-based group therapy for moral injury called “Accepting Moral Pain and Suffering for Healthcare Providers” in 7 90 min sessions	No	Feasibility (referrals, eligibility, retention, participation engagement) was strong (8 out of 10 participants; 80% vs. desired >70% eligibility) and overall, 80% of participants completed 71% of the intervention
Farrel D et al. [53]	FL HCWs and first responders	Treatment session using Blind 2 Therapist EMDR therapy scripted protocol as videoconference psychotherapy	Yes (delayed treatment intervention)	No difference in ITQ between intervention and control
Yurtsever A [55]	FL HCWs	EMDR Recent Traumatic Episode Protocol	No	Significant difference in IES between the pre-test, post-test, and follow-up test mean scores of frontline professionals group (F (1.697, 140,815) = 168,658, *p* < 0.001)
Kanstrup M et al. [56]	FL HCWs with at least two intrusive memories of work-related trauma in the week	Imagery-competing task intervention	Yes (delayed treatment intervention)	Intervention significantly reduced intrusive memory frequency compared with control (intervention median = 1.0 (IQR = 0–3), control median = 5.0 (IQR = 1–17); *p* < 0.0001, IRR −0.30; 95% CI = 0.17–0.53)
Pihlgren SA et al. [58]	FL HCWs	A brief memory reminder cue, then Tetris^®^ gameplay using mental rotation instructions for 20 min	No	Perceived as acceptable
McCann B et al. [59]	FL HCWs	Self-guided CBT, 1:1 psychological therapy with trainee psychologists	No	Perceived to be of high quality and perceived to have improved mental health and well-being
Viswanathan R et al. [60]	FL HCWs	Recurring peer support groups via telehealth led by psychiatrists	No	NR
Kubickova V et al. [57]	FL HCWs	Guided imagery-competing task intervention involving a trauma reminder cue and Tetris gameplay	No	Decrease (59%) in the mean number of intrusive memories per day from baseline (mean 1.29, SD 0.94) to postintervention (mean 0.54, SD 0.51)
Sağaltıcı E et al. [60]	FL HCW who presented to psychiatry clinic and had a positive PTSD screen	EMDR therapy, recent Traumatic Episode Protocol	No	Decline in both BDI (*p* < 0.001) and BAI (*p* < 0.001)
Bureau R et al. [62]	FL HCWs	Internet-based CBT program called My Health Too	No	>70% of participants completed at least 2/3 of the videos and were satisfied with the website
Rizzi D et al. 2025 [64]	FL HCWs	DBT	Yes (no intervention)	NSESS scores decreased in control and intervention group; no difference between groups
Gupta S et al. [69]	FL HCWs	Telehealth counseling	Yes (PE)	No significant difference between the two groups (no group effect) or group-by-time interaction (group × time effect) regarding the change in the mean scores on DASS-21 or IES-R (t = −1.65 to 0.31; *p* = 0.75 to 0.11)
Wild J et al. [71]	FL HCWs	Coaching prioritizing trigger discrimination for re-experiencing symptoms via 6 telephone sessions delivered over 6–8 weeks	No	For PCL-5 and PHQ-9, reliable recovery rates increased substantially from symptom monitoring (PCL-5: 14.6%; PHQ-9: 15.8%) to intervention (PCL-5: 77.1%; PHQ-9: 64.3%), with sustained recovery at three months (68.8% and 52.6%, respectively). Overall, rates of improvement increased from 20.0% to 63.1% for depression during intervention
Kameno Y et al. [73]	FL nurses who were high risk based on K6 score	2 therapy sessions	Yes (no intervention)	Improvement of psychological distress in the nurses identified as high-risk who received psychotherapy compared with those who did not receive psychotherapy (*p* < 0.001, mean difference = 9.80, 95% CI = 1.92 to 17.68, Cohen’s *d* = −1.50)
Castro Ribeiro T et al. [79]	FL HCWs	5 weekly sessions of heart rate variability biofeedback	No	Difference between start and end of study in PHQ-9 3.38 (*p* = 0.003), STAI T 11.52 (*p* < 0.001), and PCL-5 11.25 (*p* = 0.001)
Xu J et al. [100]	FL HCWs	Chat network counseling in person, group, WeChat (phone)	No	Significant decrease in SCL-90
Gálvez-Herrer M et al. [102]	FL HCWs	In person vs. telehealth counseling	No	553 sessions; The mean general satisfaction with the intervention was high and 96.2% (n = 252) of the participants would recommend it in future
Khairallah GM et al. [104]	FL HCWs	Employee assistance program offered free counseling	No	56 individual and 5 group sessions were held
Vagedes J et al. [113]	FL HCWs	Measured HR variability in total and in groups that mentioned emotional and spiritual key words	No	65.8% expressed ESKs. The RMSSD increased only in the ESK-expressing group (*p*< 0.001)
Kevern T et al. [126]	FL physician trainees	Residents were scheduled for a therapy appointment at no cost and could opt out	No	Needed 0.15FTE for program implementation. Attended by 59%. Feedback: 98% would recommend, 94% felt that program cared

Abbreviations: NR: not reported; FL HCWs: front-line healthcare workers; CBT: cognitive behavioral therapy; EMDR: Eye Movement Desensitization and Reprocessing; ITQ: International Trauma Questionnaire; IES-R: Impact of Events Scale Revised; BDI: Beck Depression Inventory; BAI: Beck Anxiety Inventory; IQR: interquartile range; IRR: incidence rate ratio; CI: confidence interval; SD: standard deviation; PTSD: post-traumatic stress disorder; DBT: dialectical behavioral therapy; DASS-21: Depression Anxiety Stress Scale; K6: Kessler Psychological Distress.

**Table 7:** Mindfulness: meditation, yoga, and similar interventions.

Reference	Subjects	Description of Intervention	Control Group Y/N	Results
Zhan J et al. [21]	FL HCWs	2 weeks of tai chi training	Yes (relaxation training)	Tai chi group had lower scores on PSQI (*p* < 0.05) and BAI (*p* < 0.01)
Li JM et al. [24]	FL HCWs	Brief mindfulness meditation nightly for 2 weeks	Yes (no intervention)	No significant differences in the PHQ-9, GAD-7, PSS, and AIS scores between the intervention and control groups
Pratt EH et al. [23]	FL HCWs	4-week self-directed mindfulness app with daily audio and video mindfulness content	No	28% completed >/= 1 session per week; 19% completed >75% of sessions
AlQarni AM et al. [29]	FL HCWs	2-week virtual intervention with a brief mindfulness intervention	Yes (progressive muscle relaxation)	Greater improvement in WHO-5 in intervention vs. group (81.3% vs. 51.8%, *p* < 0.01
Arts-de Jong M et al. [28]	FL HCWs	Adjusted therapist-assisted Mindfulness-based Stress Reduction group intervention	Yes (self-guided mindfulness-based intervention)	No significant difference in PHQ-SADS (mean difference (SE), 0.23 (1.03), *p* = 0.82 between intervention and control
Misra P et al. [39]	FL HCWs	Yoga sessions 3 times per week for 12 weeks	No	Post-intervention DASS-21 score decreased and WHO-5 increased significantly
Dyer NL et al. [43]	FL HCWs	20 min remote Reiki sessions on 4 consecutive days	No	Statistically significant decreases in stress (M = −2.33; *p* < 0.001), anxiety (M = −2.79; *p* < 0.001) and pain (M = −0.79; *p* < 0.001), and significant increases in well-being (M = −1.79; *p* < 0.001) and sleep quality (M = −1.33; *p* = 0.019
Reyes AT et al. [47]	FL nurses	6 weeks of mindfulness- and acceptance-based smartphone app intervention	Yes (no intervention)	AAQ-II score improved in intervention group (*p* < 0.001); RRS score improved at week 6 (*p* < 0.001); no change in mindfulness or resilience
Luo LX et al. [50]	Nurses providing COVID-19 testing	Mindfulness decompression therapy	Yes (routine psychological nursing intervention)	SCL-90 scores improved more in intervention group; improved MBI-GS scores in intervention group emotional exhaustion (1.880 ± 0.637, 0.942 ± 0.575); professional effectiveness (1.036 ± 0.888, 4.044 ± 1.087); cynicism (2.992 ± 0.901, 0.469 ± 0.494), all *p* < 0.05
Hu Y et al. [57]	FL HCWs	Internet and music therapy	Yes (routine psychological support)	Lower anxiety proportion after the intervention compared to before, 51.7% vs. 8.3%, and lower depression proportion, 38.3% vs. 5%
Lefèvre H et al. [61]	FL HCWs	The Bubble: a program and a space for relaxation and support for hospital caregivers by hospital caregivers	No	NR
Bazan GN et al. [58]	FL nurses	6-week mindfulness toolkit with in-person guided 1.5 h mindfulness, yoga, and spirituality training, 6-week daily guided reflection book that incorporated spirituality and mindfulness and meditation with accompanying nature illustrations	No	Statistically significant effect improvement in emotional exhaustion (Wilks Λ = 0.66; *F*_1,4_= 19.0; *p* = 0.001; η^2^ = 0.31), depersonalization (Wilks Λ = 0.70; *F*_1,41_= 7.93; *p* = 0.007; η^2^ = 0.16), and stress (Wilks Λ = 0.81; *F*_1,41_ = 8.81; *p* = 0.005; η^2^ = 0.17)
Sarwal R et al. [59]	FL HCWs	30 min of yoga in the morning and 15 at night x 28 days delivered via video conference	Yes (no intervention)	Intervention group had a substantially lower perceived stress at post-test at the end-line in comparison to the control group (*p*-value: 0.028). WHO Quality-of-Life psychological domain increased significantly (*p* = 0.019)
Azizoddin DR et al. [65]	FL HCWs	8 individual or group sessions of transcendental meditation over 3 months	No	Significant reduction in symptoms of depression, anxiety, stress, and sleep disturbance (*p* values < 0.001; Cohen’s *d* = 0.70–0.87)
Bigman-Peer N et al. [68]	FL HCWs	One session of shiatsu massage and reflexology	Yes (no intervention)	Reduction in burnout scores in intervention group vs. control, primarily due to an enhanced sense of accomplishment (*p* = 0.023)
Bonetto C et al. [75]	FL nurses	7-week mindfulness and compassion course	Yes (no intervention)	No significant change in PHQ-9, GAD-7, and IES between groups
Beverly E et al. [76]	FL HCWs	3 min Tranquil Cinematic-Virtual Reality simulation of a nature scene	No	Significant reduction in subjective stress scores from pre- to post-simulation (mean change = −2.2 ± 1.7, *t* = 12.749, *p* < 0.001), with a Cohen’s *d* = 1.08
Rodriguez-Vega B et al. [80]	FL HCWs	5–10 min of mindfulness practices delivered twice daily by experienced psychiatrists, psychologists, and mental health nurses	No	Sessions were perceived as being helpful with a mean rating of 8.4 on a scale from 0 to 10. Only 3 people (2%) reported a minor adverse effect (increased anxiety or dizziness)
Gunjiganvi M et al. [84]	FL HCWs	Yoga nidra daily for 2 weeks	Yes (relaxation to music daily for 2 weeks)	PHQ-9 scores decreased significantly (5.17 ± 4.25 to 3.03 ± 2.40, *p* = 0.002) compared to the Relaxation-to-Music Group (5.68 ± 4.73 to 4.34 ± 2.90, *p* = 0.064); GAD-7 scores decreased significantly in the Yoga Nidra Group (4.93 ± 3.27 to 2.33 ± 2.56, *p* < 0.001) compared to the Relaxation-to-Music Group (4.84 ± 3.94 to 4.03 ± 3.56)
DiGalbo RT et al. [85]	FL HCWs in ICU	Lavender oil applied at least once during beginning of ICU shift	No	Improvements in the MBI personal accomplishment subscale (mean (SD), 3.86 (0.81) before vs. 4.14 (1.01) after intervention; *p* = 0.04). Improvements in depersonalization were not significant. Most participants were satisfied (n = 23 (67.6%)) and compliant (n = 23 (67.6%)) with the intervention
Evans LV et al. [86]	FL EM residents and faculty	3 h virtual educational simulation intervention consisting of four simulation scenarios (CRI:SIS) with varying severity of COVID-19 presentations	No	Post-CRI:SIS, there was no significant mean difference between the groups (−1.64 points, 95% CI, −4.58 to 1.30; *p* = 0.27)
West K et al. [88]	FL HCWs	Participants drew graphic art related to identity/community and pandemic narrative	No	NR
Osman I et al., 2022 [90]	FL HCWs	4 weekly 1 h sessions of mindfulness and then participants provided pictures reflecting the theme of the mindfulness exercise that week	No	HCPs felt calmer, more competent and more compassionate
Gao F et al. [94]	FL physicians	6-week exercise routine	No	SDS decreased from 54 to 46 (*p* < 0.05); SAS decreased from 44 to 40 (*p* < 0.05)
Tran K et al. [99]	FL ICU nurses	Provide unit-based mindfulness through aromatherapy wellness program with self-reported stress experience	No	Perceived Stress Scale-10 score decreased by 9.36% and the World Health Organization-Five score increased by 10.98%. Most participants (82%) enjoyed the program, and 68% intended to continue mindfulness or aromatherapy
De Kock JR et al. [101]	FL HCWs	3 groups randomized to smartphone well-being app vs. evidence-based app using CBT vs. control	Yes (none)	Greater improvements in depression, anxiety, well-being, MT, and gratitude among participants in the digital intervention groups (MPS and NHSWBP) postintervention compared to the WL group
Romano D et al. [103]	FL HCWs	PE group workshops via Zoom	No	Based on qualitative feedback, workshop sessions were well-received
Hsieh CW et al. [105]	FL ICU nurses	6-week online program with 90 min weekly sessions on mindfulness-based stress reduction	Yes (none)	Compared with the control group, the intervention group exhibited significantly greater reductions in fatigue at T2 (*p* = 0.007) and T3 (*p* < 0.001), as well as greater improvements in resilience at T2 (*p* < 0.001) and T3 (*p* < 0.001)
Wang D et al. [107]	FL ICU nurses	Mindfulness modules: 3 groups iACT linear (12 sessions), iACT loop (constant change and flow based on Daoism), waitlist	Yes (waitlist)	Intervention had significant improvement on OCS (*t* = −38.235, *p* < 0.001), PF (*t* = 28.156, *p* < 0.001) and SQ (t = −16.336, *p* < 0.001). There were significant differences between the linear group and loop group on the PF in T2 (*t* = −8.271, *p* < 0.001), T3 (*t* = −8.366, *p* < 0.001), T4 (*t* = −8.302, *p* < 0.001), with the iACT loop model (Cohen’s *d* = 1.652) showing a slight advantage over the iACT linear model (Cohen’s *d* = 1.134)
Yin Q et al. [108]	FL HCWs	Mindfulness-based stress reduction exercise therapy	Yes (none)	After the intervention, the intervention group had significantly lower scores in the SCL-90 and the PSS (*p* < 0.05)
Hung CL et al. [114]	FL nurses	Bergamot essential oil was diffused over 4-week period	No	Only significant changes in mean LF by 25.75% (*t* = −9.69, *p* < 0.001) and mean HF by 25.75% in ICU group; BP/HR not significant
Kim S et al. [116]	FL HCWs	4-week course, online, focus on resiliency and home techniques	No	Resiliency significantly increased after the program compared to baseline, and the improvement persisted at the 1-month follow-up in both unadjusted and adjusted models (*p* < 0.05)
Lee SK et al. [119]	FL HCWs who are parents	App-delivered mindfulness exercises with smartwatch: groups included: 1) audioguided mindfulness activity; 2) in-app push prompting activity; 3) none	Yes (none)	60.4% of the participants completed both EMA1 and EMA2 on all 30 study days
Castillo-Sánchez G et al. [121]	FL HCWs	online mindfulness course	No	74% rated the course as good; 94% were satisfied with the course
Adair KC et al. [125]	FL HCWs	Web-based self-compassion tool	No	Significant improvement in emotional regulation (*p* < 0.0001) scores but not in emotional thriving
Nijland JWHM et al. [128]	FL nurses	10 min Vrelax session looking at relaxing scenes	No	PSS-10: difference 14pts: SD 13.3, *t* = 8.6 (*p* = 0.0005), 95% (10.8–17.3)
Cepeda-Lopez AC et al. [131]	FL nurses	36 mind–body based micropractices	Yes (Non-FL nurses)	No change in subjective well-being and resilience
DeTor NR et al. [132]	FL HCWs	3 videos lasting 12–19 min with didactics, exercise concentrating on mindfulness, mentalization, and self-compassion	No	Resilience levels significantly increased at 2 months (*t* = 2.88, *p* = 0.010). Decreases in emotional distress at 1 month (*t* = 3.09, *p* = 0.004) and 2 months (*t* = 2.97, *p* = 0.009)
Kanchibhotla D et al. [133]	FL HCWs	Yoga course online with breathing techniques. Total 8 h over 4 days	No	Reduction in DASS-21 scores (*p* < 0.001)
Fiol-DeRoque MA et al. [134]	FL HCWs	Self-managed and self-guided psychoeducational mobile-based intervention. no therapist	Yes (control contents on application)	Reduction in DASS-21 at 2 weeks (standardized mean difference −0.04; 95% CI −0.11 to 0.04; *p* = 0.15)
Gheradrdi-Donado ECDS et al. [135]	FL nurses	8 two-hour sessions over 8 weeks completed by mindfulness consultants online	No	Perceived stress 29.5 to 22.5 (*p* < 0.001), Mindfulness (MAAS) 47 to 55, *p* < 0.001
Hsieh HF et al. [136]	FL nurses	7 sessions in 2 days. Smartwatch given to both groups, but one had therapy.	Yes (none)	Significant reduction in stress after intervention with gong meditation (T1, β = −59.33; T2, β = −35.81; T3, β = −48.26; T4, β = −46.18; T5, β = −31.51; T6, β = −39.02; T7, β = −28.72).
Ibrahim K et al. [137]	FL nurses	Mindfulness breathing meditation twice a week for approximately 15 min per session	Yes (none)	Intervention group had improved well-being scores post- compared to pre-intervention; control group had no change
Keng SL et al. [138]	FL nurses	Headspace APP used for 3 weeks with one phone call at week 1	Yes (engaging in Lumosity games)	At one month, significant indirect effect of changes in self-compassion on depressive symptoms (indirect = −1.20, SE = 0.61, 95% CI (−2.61, −0.23)). Fear of COVID-19 decreased
Nestor MS et al. [139]	FL HCWs	Restful alertness state created by certified instructors in person or online; 10 sessions over 12 weeks	Yes (none)	*p*-values for between-group differences in change from baseline showed improvement in all scales at 3 months
Nourian M et al. [140]	FL nurses	Mindfulness-based stress reduction	Yes (none)	Statistically significant differences between intervention and control groups in sleep latency and subjective sleep quality
Osman I et al., 2021 [141]	FL HCWs	Mindfulness sessions led by 2 certified instructors. Four-week building curriculum on Zoom	No	PSS: 21.1 (SD 6.83) to 15.26 (SD 5.38). *p* < 0.001. Mindful Attention Awareness Scale (MAAS): mean increased from 3.5 to 3.9, *p* < 0.00
Prado K et al. [142]	FL HCWs	Application that uses binaural beats technology combined with sounds and frequencies that relax the mind.	No	Significant decrease in perceived stress from 19.46 ± 6.14 at pretest to 5.79 ± 6.93 at posttest (*p* < 0.001)
Thimmapuram J et al. [143]	FL physicians and advanced practice providers	Audio-guided heartfulness meditation for 6 min twice daily	Yes (none)	Significant decrease in UCLA loneliness score and PSQI in intervention group but not in control
Vajpeyee et al. [144]	FL HCWs in ICU	Yoga modules and instrumental music	Yes (none)	Positive impact of Yoga and music intervention in participants on depression, anxiety, and stress levels or scores on participants with normal as well as abnormal DASS-42 scores in a statistically significant manner
Yildirim D et al. [145]	FL nurses	30 min session with breathing techniques and relaxing music followed by discussion	Yes (none)	Significant improvements in stress, work-related strain, and psychological well-being in intervention compared to control
Dincer B et al. [148]	FL nurses	Emotional freedom technique online program	Yes (none)	Significant decrease in STAI-I and SUD in intervention compared to control
Miyoshi T et al. [153]	FL HCWs	Weekly yoga	No	Pulse: (before, after the first program: 72, 65 bpm, *p* < 0.05; before, after the last program: 75, 66, *p* < 0.05). Salivary cortisol: before: 0.077 (0.057–0.098); after last: 0.07 (0.062–0.074), *p* = 0.57.
Chang J et al. [154]	FL emergency medicine residents	Platform allowed for residents and attending physicians to acknowledge individual residents for their achievements through public praise and the granting of points that can be redeemed for rewards	No	No statistically significant change over 6 months in SPFI
Giordano F et al. [155]	FL HCWs	Unique playlist created by specialist to balance the following: favor relaxation and reduce anxiety and stress (Breathing PL), to recover energy and support concentration (Energy PL), to release tension and instill calm and peace of mind (Serenity PL)	No	Reductions in tiredness, sadness, fear, and worry were statistically significant (*p* < 0.05)
Sockalingam S et al. [156]	FL HCWs	ECHO-CWC: two 1 h sessions per week and included the following components: introductions, a mindfulness exercise, COVID-19 information question and answer, a library update on resources, a didactic presentation based on the curriculum topic, case-based discussion	No	High mean satisfaction scores for the first five sessions (4.26)
Othman SY et al. [157]	FL nurses in ICU	Eight 2.5 h MBI sessions based on self-compassion led by trained instructor	Yes (none)	Significant improvement in intervention group compared to control in MBI
Iyadurai L et al. [158]	FL HCWs in ICU	25 min cognitive task to disrupt visual mental imagery of traumatic events. 1) Brief trauma reminder cue. 2) Tetris using mental rotation instructions.	Yes (delayed intervention)	Fewer intrusive memories during week 4 in the immediate (median = 1, IQR = 0–3, n = 43), compared to the comparator delayed arm (median = 10, IQR = 6–17, n = 43), IRR 0.31, 95% CI: 0.20–0.48, *p* < 0.001. After crossover, the delayed arm also showed a significant reduction in IMs at week 8 compared to week 4

Abbreviations: NR: not reported; MBI: mindfulness-based intervention; BAI: Beck Anxiety Inventory; AIS: Athens Insomnia Scale; PSS: Perceived Stress Scale; AIS: Athens Insomnia Scale; PHQ-9: Patient Health Questionnaire-9; GAD-7: Generalized Anxiety Disorder-7; WHO-5: World Health Organization-5 well-being index; PHQ-SADS: Patient Health Questionnaire Somatic, Anxiety, and Depressive Symptoms; DASS-21: Depression Anxiety Stress Scale; AAQ-II: Acceptance and Action Questionnaire; RRS: Ruminative Responses Scale; SCL-90: Symptom Checklist-90; MBI-GS: The Maslach Burnout Inventory—General Survey; bpm = beats per minute.

**Table 8:** Studies using multiple/combined interventions.

Reference	Study Population	Description of Intervention	Control Group Y/N [Treatment in Control Arm]	Results
Gao Q et al. [8]	New ICU staff	Online program with music therapy, sleep hygiene education, psychoeducation, and relaxation training	Yes (routine wellness care)	No difference in DASS-21; improvement in secondary outcome ISI score
Rolling J et al. [17]	All FL HCWs	Intervention included support hotline, relaxation rooms, mobile teams, psychoeducation	No	170 hotline calls, 2120 relaxation room visits, 1200 contacts by mobile team
He C et al. [22]	Nurses	3-part program administered before, during, and after isolation ward consisting of individual counseling, PE, crisis hotline	No	Significant reduction in GAD-7, PHQ-9, and SSS at time 3 vs. time 1 and 2
Bernstein CA et al. [26]	FL HCWs	Psychoeducational resources, phone support line, staff support centers, clinical treatment program, team support sessions, peer support outreach, mental health and wellness programs and clergy support	No	The most heavily used service during the pandemic was the staff support centers, and the least used service was the clergy support
Agarwal AK [12]	FL HCWs	Cobalt online mental health platform offering online evidence-based tools, coaching, therapy options and asynchronous content	No	43,308 independent user sessions; 4418 appointments were scheduled with mental health counselors and clinicians
Sanford J et al. [54]	Female FL HCW	Screening, linkage to counseling or psychiatry services, leadership development, community pods, and parenting forums were designed	No	NR
Gray M et al. [13]	FL HCWs	Mental Health Liaisons, who provided preventative support to COVID-19 hospital units and Emergency Departments, and Mental Health Crisis Response Teams, who staffed 24/7 crisis response lines	No	77 calls in 3 months; Mental Health Liaisons conducted a total of 1090 contacts, which led to 38 referrals for outpatient mental health care
Wang CC et al. [66]	Case report of 1 FL nurse	Two-step telehealth intervention: (1) self-directed narrative writing vs. medical music; (2) prolonged exposure therapy vs. psychotherapy using videoconferencing	No	CAPS-5, QIDS, SDS, and PSQI declined after treatment
Appelbom S et al. [67]	FL HCWs	PE, peer support, psychologist-supervised and unsupervised group sessions, on-boarding for transferred staff, manager support, and individual sessions for workers experiencing strong stress reactions.	No	Peer support initiatives and daily group sessions were the most appreciated forms of psychological support
Korman MB et al. [78]	FL HCWs	Social Support, Tracking Distress, Education And Discussion, communitY (STEADY) program including peer partnering, wellness assessments, PE, and peer support	No	Feedback was virtually entirely positive. Requests for support were received from approximately twenty non-target units based on positive word-of-mouth, indicating program acceptability and success
DeVylder EK et al. [81]	FL HCWs	Free online public MH mobile apps; Manage Stress (interactive coping tools); Learn (psychoeducation about wellness/health); Mood Check (validated measures to track depression, anxiety, and PTSD symptoms, and well-being); and Find Resources (mental health support)	No	Participants endorsed high ratings of the acceptability, appropriateness, feasibility, knowledge, and perceived usefulness of the app.
Samadi S et al. [82]	FL HCWs in anesthesia department	Providing financial support for the attending staff to procure personal protective equipment, rearrangement of work schedules to reduce the workload, holding training sessions in virtual meetings, and improving the social network system for reducing burnout	No	NR
Rubin EW et al. [83]	FL HCWs	PE videos and peer support through synchronous video presentations, one-on-one peer support, and resourcing and referral	NR	All respondents agreed with the statement “attending the cAIR video presentation was a good use of my time” and either agreed or strongly agreed with the statement “cAIR should be available to all Swedish caregivers”
Leland LE et al. [96]	FL nursing home administrators	MH services, childcare, flexibility, and compensation	No	63 of the 94 nursing homes reported strategies in at least 1 of the 5 categories; 19 of the 36 (53%) high quality nursing homes reported strategies
Zador L et al. [117]	FL physicians	Well-being initiative including flexible time schedules, enhanced night coverage, 12 protected hours for community service	No	.05 decrease in the well-being index sum score for every 1 month of time (coefficient −0.05, 95% CI −0.01, −0.08, *p* = 0.013)
Maunder RG et al. [151]	FL HCWs	Psychological surveys self-administered at intervals (the PSAF intervention), and the availability of in-person peer support by Peer Resilience Champions (the PRC intervention)	Yes (peer-to-peer support)	Among 538 staff, there was a small but significant beneficial effect of PSAF over time (*p* = 0.01)

Abbreviations: NR: not reported; DASS-21: Depression Anxiety Stress Scale; ISI: Insomnia Severity Index; PE: psychoeducation; PHQ-9: Patient Health Questionnaire-9; GAD-7: Generalized Anxiety Disorder-7; SSS: Social Support Scale; CAPS-5: CAPS-5: Clinician-Administered PTSD Scale for DSM5; QIDS: Quick Inventory of Depressive Symptomatology; SDS: Zung Self-Rating Depression Scale; PSQI: Pittsburgh Sleep Quality Index; MH: mental health.

**Table 9:** Medication-focused studies.

Reference	Study Population	Medication Applied	Control Group Y/N [Treatment in Control Arm]	Results
Back AL et al. [7]	FL HCWs with depressive symptoms	Psilocybin 25mg	Yes (niacin)	Greater decrease in MADRS in psilocybin group compared to niacin group: −12.00 (95% CI, −17.67 to −6.33; *p* < 0.001)
Lewis BR et al. [27]	FL physicians and nurses with depression	Psilocybin-assisted psychotherapy	Yes (mindfulness intervention)	Greater decline in QIDS-SR-16 score than the MBSR-only arm from baseline to 2 weeks post-intervention (between-groups effect = 4.6, 95% CI (1.51, 7.70); *p* = 0.008)
Souza JDS et al. [35]	FL HCWs	300 mg of CBD for 28 days followed by 4 weeks after discontinuation	Yes (no medication)	Compared to control group, a significant reduction in GAD-7 delta score in the CBD group on weeks 2, 4, and 8 (Within-Subjects Contrasts, time–group interactions: F_1–125_ = 7.67; *p* = 0.006; η_p_^2^ = 0.06; F_1–125_ = 6.58; *p* = 0.01; η_p_^2^ = 0.05; F_1–125_ = 4.28; *p* = 0.04; η_p_^2^ = 0.03, respectively). CBD plasma levels were significantly associated with the reduction in clinical anxiety
Robison R et al. [49]	FL HCWs with burnout or post-traumatic stress symptoms	1 preparation session, 3 ketamine sessions, 2 integration sessions	No	Improvement in PCL-5 by 59%, PHQ-9 by 58%, and GAD-7 by 36% in post- compared to pre-treatment
Crippa JAS et al. [95]	FL HCWs	CBD 150 mg bid	Yes (supportive care)	Significant effects of group (F1.00–116.00 = 13.17; *p* < 0.001) and time–group interaction (F3.37–390.31 = 5.51; *p* < 0.001) on PHQ-9 scores; significant effects of group (F1.00–116.00 = 15.34; *p* < 0.001), and time–group interaction (F3.35–388.43 = 5.31; *p* = 0.001) on GAD-7 score; no significant change in PCL-5

Abbreviations: FL HCW: front-line healthcare worker; MADRS: Montgomery–Asberg Depression Rating Scale; PHQ-9: Patient Health Questionnaire-9; GAD-7: Generalized Anxiety Disorder-7; QIDS-SR-16: Quick Inventory of Depressive Symptomatology [16 item]; PCL-5: PTSD Checklist for DSM-5.

## Data Availability

No new data were created or analyzed in this study. Data sharing is not applicable to this article.

## Supplementary Materials

The following supporting information can be downloaded at: https://www.mdpi.com/article/10.3390/ijerph23081082/s1, Table S1: PRISMA 2020 Checklist [163].

## Appendix A. Newcastle–Ottawa Scale for Non-Randomized Studies

**Study**	**Study Type**	**Selection**				**Comparability [Stars, N]**	**Outcome [Stars, N]**			**Final Score**	**Risk of Bias**
		**Representativeness**	**Selection of Non-Exposed**	**Ascertainment of Exposure**	**Outcome Not Present at Start**	**Comparability on the Basis of Design or Analysis**	**Assessment of Outcome**	**Follow-Up Duration**	**Adequacy of Follow-Up**		
Rizzi D et al., 2022 [9]	cohort	*								1	Poor
Rolling J et al. [17]	cohort	*								1	Poor
Zingela Z et al. [18]	cohort	*						*	*	3	Poor
Cheng P et al. [19]	cohort	*								1	Poor
Robles R et al. [20]	cohort	*			*				*	3	Poor
He C et al. [22]	cohort	*							*	3	Poor
Bernstein CA et al. [26]	cohort									0	Poor
Malik M et al. [30]	cohort	*								1	Poor
Blake H et al. PMID 32357424 [31]	cohort	*								1	Poor
Trottier K et al. [32]	cohort	*			*			*	*	3	Poor
Costello Z et al. [33]	cohort	*						*		2	Poor
Captari LE et al. [34]	cohort	*								1	Poor
LeGoff DB et al. [36]	cohort	*			*				*	3	Poor
Thum CC et al. [37]	cohort	*								1	Poor
Garcia-Vazquez B et al. [38]	cohort	*							*	2	Poor
Misra P et al. [39]	cohort	*						*	*	3	Poor
Torricelli L et al. [40]	cohort	*									Poor
Ghazanfarpour M et al. [41]	cohort	*	*						*	3	Poor
O’Keefe VM et al. [42]	cohort	*						*		2	Poor
Dyer NL et al. [43]	cohort	*								1	Poor
Feinstein RE et al. [44]	cohort	*								1	Poor
Gayed A et al. [45]	cohort	*							*	2	Poor
Smith-MacDonald L et al. [46]	cohort	*							*	2	Poor
Agarwal AK [12]	cohort	*								1	Poor
Robison R et al. [49]	cohort	*							*	2	Poor
Luo LX et al. [50]	cohort	*	*						*	3	Poor
McLean CP et al. [52]	cohort	*								1	Poor
Sanford J et al. [54]	cohort									0	Poor
Yurtsever A [55]	cohort	*								1	Poor
Hu Y et al. [57]	cohort	*	*						*	3	Poor
Pihlgren SA et al. [58]	cohort	*								1	Poor
McCann B et al. [59]	cohort	*								1	Poor
Viswanathan R et al. [60]	cohort	*								1	Poor
Lefèvre H et al. [61]	cohort	*								1	Poor
Zhang N et al. [56]	cohort	*							*	2	Poor
Kubickova V et al. [57]	cohort	*							*	2	Poor
Bazan GN et al. [58]	cohort	*							*	2	Poor
Sarwal R et al. [59]	cohort	*	*			*			*	4	Fair
Sağaltıcı E et al. [60]	cohort	*			*				*	3	Poor
Geoffroy PA et al. [6]	cohort	*								1	Poor
Gray M et al. [13]	cohort	*								1	Poor
Bureau R et al. [62]	cohort	*								1	Poor
Cole CL et al. [63]	cohort	*								1	Poor
Azizoddin DR et al. [65]	cohort	*							*	2	Poor
Wang CC et al. [66]	cohort	*								1	Poor
Appelbom S et al. [67]	cohort	*								1	Poor
Bigman-Peer N et al. [68]	cohort	*							*	2	Poor
Fung K et al. [70]	cohort	*								1	Poor
Wild J et al. [71]	cohort	*			*				*	3	Poor
Ache ALDS et al. [72]	cohort	*								1	Poor
Kameno Y et al. [73]	cohort	*			*				*	3	Poor
Bonetto C et al. [75]	cohort	*							*	2	Poor
Beverly E et al. [76]	cohort	*							*	2	Poor
Blake H et al. PMID 33333913 [77]	cohort	*								1	Poor
Korman MB et al. [78]	cohort	*								1	Poor
Castro Ribeiro T et al. [79]	cohort	*							*	2	Poor
Rodriguez-Vega B et al. [80]	cohort	*								1	Poor
DeVylder EK et al. [81]	cohort	*								1	Poor
Samadi S et al. [82]	cohort	*								1	Poor
Rubin EW et al. [83]	cohort									1	Poor
DiGalbo RT et al. [85]	cohort	*							*	2	Poor
L’Engle K et al. [87]	cohort	*								1	Poor
West K et al. [88]	cohort	*								1	Poor
Connors JN et al. [89]	cohort	*							*	2	Poor
Osman I et al., 2022 [90]	cohort	*							*	2	Poor
Ballesio A et al. [91]	cohort	*								1	Poor
Kumar N et al. [93]	cohort	*			*	*	*			4	Fair
Leland NE et al. [96]	cohort			*		*		*	*	4	Fair
Vogt KS et al. [97]	cohort			*	*	*		*		4	Fair
Tran K et al. [99]	cohort			*				*		2	Poor
Xu J et al. [100]	cohort			*						1	Poor
Gálvez-Herrer M et al. [102]	cohort	*			*			*		3	Poor
Romano D et al. [103]	cohort		*					*		2	Poor
Khairallah GM et al. [104]	cohort	*		*				*		3	Poor
Rene R et al. [106]	cohort	*	*					*		3	Poor
Kelly F et al. [109]	cohort	*			*			*		3	Poor
Monette DL et al. [111]	cohort		*					*	*	3	Poor
Shopen N et al. [112]	cohort	*	*				*	*		5	Fair
Vagedes J et al. [113]	cohort	*					*	*	*	4	Fair
Hung CL et al. [114]	cohort				*			*	*	3	Poor
Moody KM et al. [115]	cohort		*		*		*	*		4	Fair
Kim S et al. [116]	cohort		*		*			*		3	Poor
Zador L et al. [117]	cohort				*	*		*		3	Poor
Ward MC et al. [118]	cohort				*			*		3	Poor
Castillo-Sánchez G et al. [121]	cohort	*				*				2	Poor
Cheng W et al. [122]	cohort	*	*							2	Poor
Allsopp K et al. [123]	cohort				*			*		2	Poor
Hartung K et al. [124]	cohort	*			*			*		4	Fair
Adair KC et al. [125]	cohort		*		*					2	Poor
Kevern T et al. [126]	cohort			*		*		*		3	Poor
Nijland JWHM et al. [128]	cohort	*	*		*			*		5	Fair
Al Ozairi A et al. [130]	cohort	*			*	*		*		4	Fair
Cepeda-Lopez AC et al. [131]	cohort	*	*		*	*		*		5	Fair
DeTor NR et al. [132]	cohort				*			*		2	Poor
Kanchibhotla D et al. [133]	cohort	*			*			*		4	Fair
Gheradrdi-Donado ECDS et al. [135]	cohort	*			*	*		*		4	Fair
Ibrahim K et al. [137]	cohort	*			*	*					Fair
Nestor MS et al. [139]	cohort	*	*		*	*		*		6	Fair
Osman I et al., 2021 [141]	cohort	*						*		2	Poor
Prado K et al. [142]	cohort	*						*		2	Poor
Vajpeyee et al. [144]	cohort		*		*			*		3	Poor
Teall AM et al. [149]	cohort	*	*					*		3	Poor
Sun L et al. [150]	cohort	*	*					*		3	Poor
Rosen B et al. [152]	cohort			*				*		2	Poor
Miyoshi T et al. [153]	cohort	*					*	*		4	Fair
Chang J et al. [154]	cohort	*						*		4	Fair
Giordano F et al. [155]	cohort	*				*		*		3	Poor
Sockalingam S et al. [156]	cohort	*	*					*		3	Poor

## Appendix B. Search Terms for Systematic Review

(((Stress) OR (distress) OR (burnout) OR (compassion fatigue) OR (moral injury) OR (moral distress)) OR ((trauma) OR (traumatic) OR (post traumatic stress disorder) or (PTSD)) OR ((anxiety) OR (anxious)) OR ((depress) OR (depression) OR (depressive disorder) OR (MDD)) OR ((mental health) OR (psychological) OR (psychiatric) OR (psychopathology) OR psychiatric)) AND ((SARS-CoV-2) OR (COVID-19) OR (COVID) OR (2019 novel coronavirus infection) OR (2019-nCoV infection) OR (coronavirus)) AND ((doctor) OR (nurse) OR (Physician) OR (Health personnel) OR (Healthcare provider) OR (Healthcare worker) OR (frontline) OR (healthcare staff) OR (medical staff) OR Medical worker) OR (frontline)) AND ((Psychiatry) OR (Intervention) OR (program) OR (treatment) OR (Access) OR (implementation) OR (support) OR (hotline) OR (psychologist) OR (psychiatrist) OR (psychology))

Filters: published between 1 January 2020 and 1 March 2026

Database-specific adaptations to search: none
